# Genome-Wide Association Study to Identify the Genetic Determinants of Otitis Media Susceptibility in Childhood

**DOI:** 10.1371/journal.pone.0048215

**Published:** 2012-10-25

**Authors:** Marie S. Rye, Nicole M. Warrington, Elizabeth S. H. Scaman, Shyan Vijayasekaran, Harvey L. Coates, Denise Anderson, Craig E. Pennell, Jenefer M. Blackwell, Sarra E. Jamieson

**Affiliations:** 1 Telethon Institute for Child Health Research, Centre for Child Health Research, The University of Western Australia, Subiaco, Australia; 2 School of Women's and Infants' Health, University of Western Australia, Perth, Australia; 3 Department of Otolaryngology, Head and Neck Surgery, Princess Margaret Hospital for Children, Perth, Australia; 4 Department of Otolaryngology, Head and Neck Surgery, University of Western Australia, Western Australia, Australia; University of Bristol, United Kingdom

## Abstract

**Background:**

Otitis media (OM) is a common childhood disease characterised by middle ear inflammation and effusion. Susceptibility to recurrent acute OM (rAOM; ≥3 episodes of AOM in 6 months) and chronic OM with effusion (COME; MEE ≥3 months) is 40–70% heritable. Few underlying genes have been identified to date, and no genome-wide association study (GWAS) of OM has been reported.

**Methods and Findings:**

Data for 2,524,817 single nucleotide polymorphisms (SNPs; 535,544 quality-controlled SNPs genotyped by Illumina 660W-Quad; 1,989,273 by imputation) were analysed for association with OM in 416 cases and 1,075 controls from the Western Australian Pregnancy Cohort (Raine) Study. Logistic regression analyses under an additive model undertaken in GenABEL/ProbABEL adjusting for population substructure using principal components identified SNPs at *CAPN14* (rs6755194: OR = 1.90; 95%CI 1.47–2.45; P_adj-PCA_ = 8.3×10^−7^) on chromosome 2p23.1 as the top hit, with independent effects (rs1862981: OR = 1.60; 95%CI 1.29–1.99; P_adj-PCA_ = 2.2×10^−5^) observed at the adjacent *GALNT14* gene. In a gene-based analysis in VEGAS, *BPIFA3* (P_Gene_ = 2×10^−5^) and *BPIFA1* (P_Gene_ = 1.07×10^−4^) in the *BPIFA* gene cluster on chromosome 20q11.21 were the top hits. In all, 32 genomic regions show evidence of association (P_adj-PCA_<10^−5^) in this GWAS, with pathway analysis showing a connection between top candidates and the TGFβ pathway. However, top and tag-SNP analysis for seven selected candidate genes in this pathway did not replicate in 645 families (793 affected individuals) from the Western Australian Family Study of Otitis Media (WAFSOM). Lack of replication may be explained by sample size, difference in OM disease severity between primary and replication cohorts or due to type I error in the primary GWAS.

**Conclusions:**

This first discovery GWAS for an OM phenotype has identified *CAPN14* and *GALNT14* on chromosome 2p23.1 and the *BPIFA* gene cluster on chromosome 20q11.21 as novel candidate genes which warrant further analysis in cohorts matched more precisely for clinical phenotypes.

## Introduction

Otitis media (OM) is a common disease of early childhood characterised by inflammation of the middle ear cavity. There are two related clinical phenotypes of OM; acute otitis media (AOM) and otitis media with effusion (OME). Acute OM is defined as the presence of a purulent middle ear effusion (MEE) due to acute infection and is accompanied by fever and painful inflammation of the tympanic membrane (TM). Whilst the majority of children experience at least one episode of AOM by school age, up to 40% suffer from recurrent AOM (rAOM; ≥3 episodes in 6 months or ≥4 episodes in 12 months) [1], [2]. In contrast, OME presents with a serous, non-purulent MEE which can become chronic (COME; MEE ≥3 months with no fluid free intervening period). Severe OM (rAOM or COME) can cause mild to moderate conductive hearing loss and perforation of the TM leading to possible delays in speech development and educational difficulties in school [3]. In view of this, severe OM is frequently managed via surgical insertion of tympanostomy tubes.

Whilst epidemiological analyses have identified several environmental risk factors [4] that play an apparent role in OM susceptibility, heritability studies also reveal that a significant component of OM risk is attributable to genetic factors. Three heritability studies performed using twins and triplets have been published to date with results suggesting that genetic factors contribute between 45–74% of the risk for OM [5], [6], [7]. Genome-wide linkage studies have implicated several chromosomal regions, including 10q26, 19q13.43, 3p25.3, 17q12 and 10q22.3, as harbouring susceptibility loci, but the genes underlying these linkage peaks have yet to be identified [8], [9], [10]. In addition, candidate gene studies have identified a handful of genes contributing to OM susceptibility including several immune genes, such as *IL10* and *TNF* (reviewed in [11]), as well as *FBXO11*, which has been significantly associated with OM in three independent cohorts [12], [13].

One method to identify novel genes that confer susceptibility to complex diseases such as OM is via a genome-wide association study (GWAS). In recent years, GWAS have increased in popularity with numerous loci robustly associated with various common, complex conditions [14]. However, there has been no GWAS of OM susceptibility reported to date. Here we present results from the first GWAS of OM carried out using cases and controls derived from The Western Australian Pregnancy Cohort (Raine) Study (‘the Raine Study’) [15], a longitudinal birth cohort based in Western Australia, with replication performed in the Western Australian Family Study of OM (WAFSOM) [13].

## Methods

### Study participants and phenotype definition

The Raine Study was used for a ‘discovery’ GWAS. The Raine Study is a longitudinal birth cohort of 2,868 children whose mothers were recruited from antenatal clinics in Western Australia during early pregnancy in 1989–1991 [15]. Children were initially assessed at birth and subsequently through comprehensive follow-up assessments at specific ages to the current 21^st^ year follow-up. For the purposes of this study, data collected from clinical examinations and parental questionnaires at the (average) ages of one, two and three years was used to define a phenotype for OM. In the full cohort, there were 644 cases and 2,221 controls. Three children were excluded from all analyses due to diagnosis of cleft palate, a known medical risk factor for OM susceptibility [16]. A total of 1532 Raine Study participants (428 cases and 1,101 controls) had genome-wide data available from an Illumina 660W Quad Beadchip (cf. below) and so were included in this discovery GWAS. Children (N = 279; 65%) were defined as a case if clinical examination in the first three years of life indicated presence of inflamed, retracted or scarred TM, MEE or tympanostomy tubes *in situ*. Participants (N = 245; 57%) were also classified as a case where parents reported ≥3 episodes of AOM by the age of 3 yrs; 35% qualifying on the basis questionnaire criteria alone. Children with no clinical or parental reported history of OM by the age of 3 yrs were classified as controls. Based on questionnaire data, 94% self-identified their ethnicity as Caucasian. Recruitment to the Raine Study and all follow-ups were approved by the Human Ethics Committee at King Edward Memorial Hospital and/or Princess Margaret Hospital for Children (PMH), with specific adult re-consent for DNA for those individuals participating in this study.

For replication of selected top hits, the WAFSOM was used. This is a family-based cohort that recruited probands on the basis of tympanostomy tube insertion for rAOM or COME prior to 3 years of age. Their parents and any affected siblings (defined as having ≥3 episodes of AOM by age 3 yrs and/or COME with tympanostomy tubes inserted or recommended) were also recruited. Details of this study have been previously described [13]. In total, 645 families consisting of 793 affected children (190 rAOM only; 546 rAOM and OME/COME; 57 COME only) were available for replication genotyping of which 93.2% self-reported their ethnicity as Caucasian. Recruitment to WAFSOM was approved by the Human Ethics Committee at PMH. Written, informed consents were obtained both for participation in the study and for DNA consent from all adults or from the parents of participants less than 18 years of age.

### Epidemiological analysis of potential covariates

Retrospective data collated from parental completed questionnaires at the year 1, 2 and 3 follow-up for the Raine Study provided information on potential covariates. This includes information on environmental covariates, such as day care attendance, breast feeding duration, exposure to furry pets, exposure to post-natal tobacco smoke and socio-economic status as well as relevant clinical diagnoses (i.e. reflux requiring medication, allergy and asthma) in childhood. For analysis purposes, each variable was converted into a two-tier categorical variable based on the data available in the questionnaire and analysed for association with OM using logistic regression models in STATA v10 [17].

### Sample preparation

For the majority of Raine Study participants genomic DNA (gDNA) was extracted from whole blood, collected via venapuncture utilising 4 mL K_2_EDTA vacuum tubes, from 1532 participants who attended the 14- or 17-year follow-up. Extraction of DNA from whole blood was performed utilising Qiagen PureGene chemistry. For a small subset of individuals (∼5%), gDNA was extracted from saliva using the Oragene technology (DNA Genotek, Ontario, Canada) as per manufacturer's instructions. Samples were quantified using spectrophotometry, diluted to a normalised concentration with reduced EDTA TE buffer and stored at −80°C. For the majority of WAFSOM participants, gDNA was extracted from saliva using the Oragene technology (DNA Genotek, Ontario, Canada). For a subset of WAFSOM cases (∼8%), blood samples were collected at the time of tympanostomy tube insertion and DNA was extracted from neutrophil pellets using a salting-out method [18]. WAFSOM samples were quantified using spectrophotometry, diluted in TE buffer and stored at −20°C.

### Discovery GWAS and quality control

Genotyping and quality control for the Raine Study DNAs has been described elsewhere [19]. Briefly, genome-wide data were generated at the Centre for Applied Genomics (Toronto, Ontario, Canada) using an Illumina 660W Quad Beadchip. Quality control (QC) checks were performed for individuals and single nucleotide polymorphisms (SNPs) in the discovery GWAS using PLINK [20]. Samples were genotyped in two separate batches; no batch effects were detected. Replicate samples with the lower genotyping success rate and plate controls were removed. Individuals were then excluded if they had a gender mismatch between reported gender and that determined on the basis of X chromosome data (N = 7), had a genotyping success rate <97% (N = 16), were related to other participants at the level of half-siblings or first cousins by IBD sharing (i.e. π>0.1875; N = 68) or if they had a low level of heterozygosity (i.e. h<0.3; N = 4). In total, 1,491 individuals passed QC and were available for analysis (416 cases and 1,075 controls). SNPs were excluded on the basis of deviation from Hardy-Weinberg Equilibrium (HWE P<5.7×10^−7^; N = 919 SNPs), having a genotype call rate <95% (N = 97,718 SNPs) or having a minor allele frequency <1% (N = 119,246 SNPs). A total of 535,632 SNPs passed QC checks and were available for analysis.

### Discovery GWAS analysis

After QC, principal components (PCs) analysis (PCA) was carried out in EIGENSTRAT [21] using a pruned subset of 42,888 SNPs in linkage equilibrium (pairwise r^2^≤0.1). To account for population substructure, the first two PCs have been used as covariates in association analyses. Imputation of un-typed or missing genotypes was also performed using MACH v1.0.16 for the 22 autosomes with the CEU samples from HapMap Phase2 (Build 36, release 22) used as a reference panel and r^2^>0.3 as the default threshold in MACH for accepting imputed genotypes. After imputation, 2,509,905 SNPs (i.e. 535,632 genotyped; 1,974,273 imputed) were available for analysis. Power approximations estimated for a disease prevalence of 0.3 show that the Raine Study (416 cases and 1,075 controls) has 54.6% power to detect associations at an alpha level of P = 1×10^−5^ with an effect size or odds ratio (OR) of 1.5 for SNPs with a MAF = 0.1; 76% power at P = 1×10^−4^, and 92% power at P = 0.001. Association analysis under an additive model was performed using logistic regression within GenABEL (for genotyped SNPs) or ProbABEL (imputed data) available at http://www.genabel.org/ [22], [23]. Analysis was initially performed adjusting for 2 PCs, and then repeated adjusting for 2 PCs and independently associated covariates as indicated. Nominal P-values are reported throughout. A conservative threshold of P_adj-PCA_<10^−5^ was used to identify SNPs/regions of interest for follow-up. Logistic regression modelling for independent effects between pairs of SNPs was undertaken in R version 2.15.0 (URL http://www.R-project.org/), and results presented as P_LRT_ for the likelihood ratio comparison. Gene based tests of association were performed using Versatile Gene-based Association Study (VEGAS) [24]. For gene based analysis the GenABEL single-point P_adj-PCA_-values for all SNPs were assigned to one or more genes (defined by a boundary of +/−20 kb from the 5′ and 3′ untranslated regions). Linkage disequilibrium (LD) and the number of SNPs per gene were taken into account. A total of 17,206 autosomal genes were analysed. Hence, the P-value needed to achieve statistical significance taking multiple testing into account for this gene-based analysis is P_Gene_ = 2.9×10^−6^ ( = 0.05/17,206) [24]. Regional plots of association were created using LocusZoom [25] in which -log_10_(P_adj-PCA_ values) were graphed against their chromosomal location. Pairwise LD patterns between all regional SNPs and the top SNP were calculated specifically for this Raine Study data.

### Replication analysis - Selection of Genes and SNP genotyping

Ingenuity Pathway Analysis (IPA; Ingenuity® Systems, www.ingenuity.com) was used to identify potential functional pathways based on genes in which associated SNPs from the discovery GWAS (P<10^−5^) were clustered. Replication of top hits from the discovery GWAS plus genes identified via pathway analysis was undertaken in samples from the WAFSOM cohort. Genes selected for replication were genotyped using their respective top SNPs from the discovery GWAS with at least two further haplotype tagging (tag-) SNPs (r^2^>0.8 in the CEU HapMap population) genotyped for each gene [26]. A total of 20 SNPs were selected for genotyping, which was carried out at KBiosciences (Hertfordshire, UK) using the KASPar chemistry. Analysis of HWE in the replication data was performed in STATA v10 using unaffected, unrelated individuals.

### Analysis of the Replication cohort

Power approximations using the method of Knapp [27] estimate that the 645 families within the WAFSOM cohort have 99% power to detect association with an effect size (OR) of 2 at P = 0.001 for SNPs with MAF ≥0.15. This may be an underestimation as some families contain multiple affected children (793 in total). Prior to association analysis Mendelian inconsistencies in family data were identified and removed using the PedCheck software [28]. Family-based association analysis of replication data was performed using conditional logistic regression under an additive model. For this analysis case/pseudo-control (CPC) datasets are generated in which each affected offspring is matched with one to three pseudo-controls derived from the remaining potential genotypes of the parental mating [29]. OR, 95% confidence intervals and P-values are calculated using a robust sandwich estimator of the variance and a Wald χ^2^ test statistic to control for clustering of affected siblings within families for 1df (allele-wise) tests. CPC was implemented in STATA v10.0. Patterns of LD were analysed in Haploview 4.2 [30]. As confirmation, association analyses were also undertaken using a conventional transmission disequilibrium test in the family-based association test (FBAT) [31].

## Results

### Discovery GWAS

**Figure 1:**
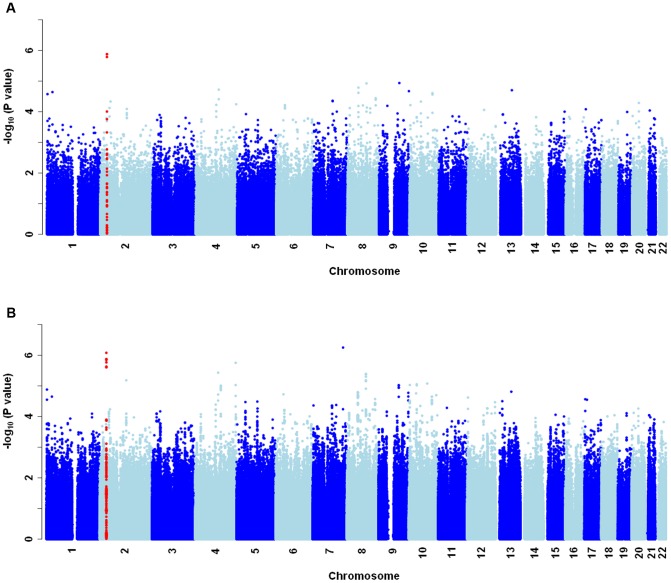
Manhattan plots for the discovery GWAS carried out in the Raine Study. Association analysis was performed on (A) genotyped SNPs, and (B) the full imputed dataset, corrected for the first two principal components to account for population stratification. Plots show −log_10_(P values) of association ordered by chromosomal location. Red dots highlight the region of the top hits CAPN14 and GALNT14 on chromosome 2p23.1.

Following quality control checks (see methods), Illumina 660W Quad Beadchip genome-wide data was available for 535,544 genotyped SNPs (2,524,817 after imputation) in 416 OM cases and 1,075 controls. Although the Raine Cohort was recruited as a predominantly Caucasian cohort, there was evidence for some level of population substructure from PCA (Figure S1A). A quantile-quantile plot of the observed versus expected −log_10_(P_adj-PCA_-value) shows no inflation of association test scores (inflation factor λ = 0.989; Figure S1B) when the first two PCs are included as covariates in the association analysis for OM. Our genome-wide analysis for OM susceptibility genes tested the additive effects of each SNP, adjusted throughout for the first 2 PCs, using GenABEL [22] for genotyped data or ProbABEL for imputed data [23]. Manhattan plots showing the genome-wide results for the genotyped and full imputed datasets are presented in Figure 1.

In the Raine GWAS analyses (Figure 1), no single variant achieved genome-wide significance, commonly accepted as P<5×10^−8^ [33]. Although we cannot be certain that associations observed were not due to type I errors, we highlight here top hits which are supported by strong biological candidacy. Genome-wide analysis adjusted for 2 PCs (Figure 1A) identified 4 genotyped SNPs showing association at P_adj-PCA_<10^−6^ and 42 genotyped SNPs showing association at P_adj-PCA_<10^−5^ (20 expected by chance at P<10^−5^) in 32 genomic regions. The top 25 genotyped SNPs from the PC adjusted analysis are summarised in Table 1. The top hit was at two polymorphisms (rs13386850 and rs13408922; OR = 1.86; P_adj-PCA_ = 1.32×10^−6^) which were in complete LD (r^2^ = 1) and located ∼4.9 kb upstream of the canonical calcium-activated neutral proteinase or calpain 14 (*CAPN14*) gene on chromosome 2p23.1. PC adjusted analysis of the full imputed dataset (Figure 1B) confirmed the *CAPN14* region as the most significantly associated region with improved evidence for association (rs6755194: OR = 1.90; 95%CI 1.47–2.45; P_adj-PCA_ = 8.3×10^−7^). Other genes of known function highlighted by the top 25 SNPs (Table 1) include *CCD27*, *LIN28*, *FBXO11*, *GRID2*, *SORBS2*, *CDK14*, *ROR2*, *SORCS3* and the psuedogene *BPIFA4* immediately upstream of functional *BPIFA3*.

**Table 1:** Top 25 genotyped polymorphisms from the discovery GWAS carried out in the Raine Study ordered by chromosome and physical location with analysis adjusted for the first two PCs using GenABEL.

SNP	Chr	Physical Location	Major/Minor	MAF	Risk Allele	Gene/Region^a^	P_adj-PCA_	OR_adj-PCA_^b^(95% CI)
rs1175549	1	3681587	A/C	0.237	A	CCDC27	2.65×10^−5^	1.56 (1.27–1.91)
rs12728900	1	26619394	G/A	0.249	A	LIN28	2.28×10^−5^	1.47 (1.23–1.75)
rs13408922	2	31298330	C/A	0.095	A	CAPN14	1.32×10^−6^	1.86 (1.44–2.38)
rs13386745	2	31299119	A/G	0.096	G	CAPN14	1.63×10^−6^	1.84 (1.44–2.37)
rs13386850	2	31299195	A/C	0.095	C	CAPN14	1.32×10^−6^	1.86 (1.44–2.38)
rs330787	2	47894881	G/A	0.358	G	FBXO11	4.64×10^−5^	1.43 (1.21–1.70)
rs11097383	4	94802863	A/G	0.158	G	GRID2	5.97×10^−5^	1.55 (1.25–1.92)
rs10008015	4	106224696	A/G	0.112	G	Intergenic	3.89×10^−5^	1.66 (1.30–2.11)
rs1859161	4	106262141	A/G	0.087	G	Intergenic	1.90×10^−5^	1.78 (1.37–2.32)
rs11940126	4	187027903	G/A	0.045	A	SORBS2	5.70×10^−5^	2.09 (1.46–3.00)
rs10242197	7	90097848	G/A	0.194	G	CDK14	4.38×10^−5^	1.59 (1.27–1.98)
rs10488001	7	90493976	C/A	0.089	A	CDK14	4.53×10^−5^	1.72 (1.33–2.24)
rs1496306	8	50703261	G/A	0.095	A	Intergenic	1.63×10^−5^	1.76 (1.36–2.28)
rs2132528	8	50768208	A/G	0.093	G	Intergenic	2.41×10^−5^	1.75 (1.35–2.26)
rs13438948	8	87927894	G/A	0.087	A	Intergenic	1.19×10^−5^	1.82 (1.39–2.39)
rs7846284	8	130439417	G/A	0.431	A	Intergenic	4.06×10^−5^	1.40 (1.19–1.65)
rs7846684	8	130439596	A/G	0.432	G	Intergenic	3.60×10^−5^	1.41 (1.20–1.65)
rs11790808	9	93628319	A/G	0.054	G	ROR2	1.15×10^−5^	2.09 (1.50–2.91)
rs10776851	9	137857942	G/A	0.134	A	CAMSAP1	2.13×10^−5^	1.63 (1.30–2.05)
rs4575213	10	53095334	C/A	0.396	C	PRKG1	4.65×10^−5^	1.41 (1.20–1.67)
rs10884043	10	106521693	A/G	0.261	A	SORCS3	2.74×10^−5^	1.53 (1.25–1.86)
rs4556466	10	106548015	A/G	0.259	A	SORCS3	2.47×10^−5^	1.53 (1.26–1.87)
rs9564897	13	71810339	C/A	0.340	A	Intergenic	1.98×10^−5^	1.43 (1.22–1.69)
rs17396317	20	31254038	G/A	0.118	A	BPIFA4P**^c^**	5.15×10^−5^	1.62 (1.28–2.05)
rs1894516	X	123961302	G/A	0.320	A	Intergenic	6.64×10^−6^	1.37 (1.19–1.57)

Where MAF  =  minor allele frequency, OR  =  odds ratio, 95% CI = 95% confidence intervals. **^a^ -** Gene/Region is based on annotation from Ensembl build 54; intergenic SNPs fall >20 kb from an annotated gene. **^b^ -** Odds ratios are shown for the risk allele; **^c^** - rs17396317 lies within the pseudogene *BPIFA4*, 15.258 kb upstream of *BPIFA3*.

**Figure 2:**
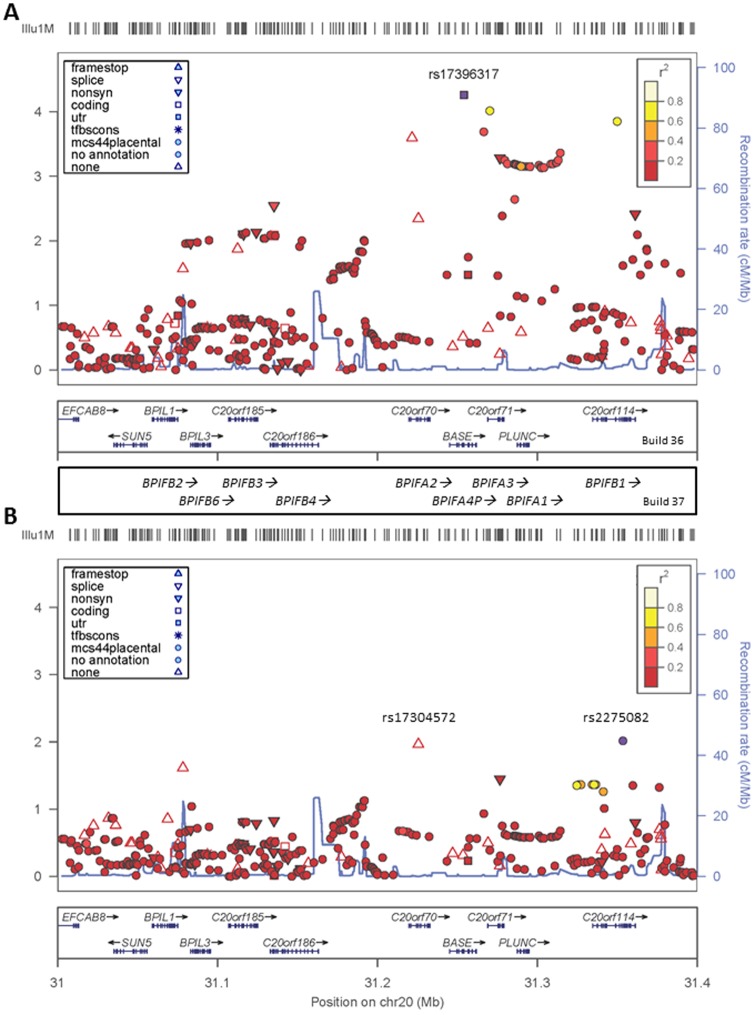
Regional LocusZoom plots of association across the region of chromosome 20 containing the *BPIFA* genes. (A) LocusZoom plots of association in the imputed dataset adjusted for PCs across the *BPIFA4P-BPIFA3-BPIFA1* gene region in the full discovery set of 1491 Raine Study participants. (B) The same region after adjusting for the top SNP rs17396317. The top associated SNP (rs17396317) is represented by purple circle in (A), the colour of all other SNPs is representative of the pairwise r^2^ value with the relevant top SNP using patterns of LD from the Raine Study cohort. Recombination rates are shown by the solid blue line. Physical positions and gene designations are based on NCBI Build 36 of the human genome. Gene designations for NCBI Build 37 are indicated in the panel between (A) and (B).

To provide in-depth gene-wide evidence for specific genes associated with OM we undertook a gene-based analysis in VEGAS using P_adj-PCA_ results from the GWAS as input data. This revealed a number of additional associated genes (Table 2), none of which withstand strict correction for the 17,206 genes interrogated (P_Gene_ = 2.9×10^−6^). However, *CAPN14* (P_Gene_ = 5.3×10^−5^) was amongst the top hits, with additional genes belonging to the BPI/PLUNC structural superfamily of palate, lung and nasal epithelium proteins also amongst the top hits; *BPIFA3* (P_Gene_ = 2.00×10^−5^) and *BPIFA1* (P_Gene_ = 1.07×10^−4^). The top SNP at *BPIFA3* is rs17396317 (Table1), lying within the pseudogene *BPIFA4* 15.258 kb upstream of *BPIFA3.* The VEGAS analysis lends support to the region of chromosome 20q11.21 carrying the cluster of BPI superfamily genes as of particular interest as an OM susceptibility locus. Figure 2A plots the fully imputed dataset across this region, highlighting the peak of association at rs17396317. Adjusting for this top SNP (Figure 2B) reduces significance (P_adj-PCA_≥0.01) across the region. Two residual signals at rs17304572 in *BPIFA2* and at rs2275082 in *BPIFB1* each provided weak evidence (P_LRT_ = 0.002 and P_LRT_ = 0.01, respectively) for independent effects when added to a model with the top SNP rs17396317. Conversely, rs17396317 provided strong evidence for independent effects when added to a model with either rs17304572 (P_LRT_ = 3.2×10^−4^) or rs2275082 (P_LRT_ = 3.02×10^−5^). The weight of evidence from the VEGAS analysis (Table 2) suggests that the etiological gene in this region is *BPIFA3* (P_Gene_ = 2.00×10^−5^), although *BPIFA1* (P_Gene_ = 1.07×10^−4^) cannot be discounted.

**Table 2:** Top 15 genes from VEGAS gene-based analysis based on GenABEL P-values from the Raine Study discovery GWAS after adjustment for PCs.

Gene	Chr	Start - End	No. SNPs	P_adj-PCA_	Top SNP
LRRC47	1	3686643–3702928	13	1.19×10^−4^	rs1175549
LIN28	1	26609855–26628806	14	3.63×10^−4^	rs12728900
PALMD	1	99884185–99932847	27	6.55×10^−4^	rs1338571
CAPN14	2	31249428–31310228	48	5.30×10^−5^	rs13408922
EHD3	2	31310706–31344764	32	4.03×10^−4^	rs13408922
MSH6	2	47863724–47887596	14	5.89×10^−4^	rs330787
CNNM4	2	96790365–96841355	6	8.87×10^−4^	rs968470
CTDSPL	3	37878672–38000964	27	1.70×10^−4^	rs6776745
VILL	3	38010081–38023680	9	2.27×10^−4^	rs11711286
RBPJ	4	25930429–26042376	18	8.94×10^−4^	rs968470
PALM2	9	111442892–111753577	208	5.60×10^−4^	rs16914575
AQR	15	32935843–33049287	30	6.81×10^−4^	rs963425
ZNF770	15	33057835–33067746	10	4.68×10^−4^	rs2060810
BPIFA3	20	31268795–31279220	8	2.00×10^−5^	rs17396317 a
BPIFA1	20	31287462–31294776	8	1.07×10^−4^	rs17305657

a - rs17396317 lies within the pseudogene *BPIFA4*, 15.258 kb upstream of *BPIFA3*.

Ordered by chromosome and physical location.

### Analysis of environmental covariates in the Raine Study cohort

One advantage of using a longitudinal birth cohort such as the Raine Study is the availability of extensive data at multiple time points on the disease phenotype and potential covariates. As outlined in the methods, Raine Study participants were defined as having OM if clinical examination at follow-up in the first three years of life indicated presence of inflamed, retracted or scarred TM, MEE or tympanostomy tubes *in situ*, or if parents reported ≥3 episodes of AOM by the age of 3 yrs. Participants with no clinical or parental reported history of OM by the age of 3 yrs were classified as controls. Previous studies have identified several environmental and clinical variables as potentially influencing OM risk, including duration of exclusive breast feeding, day care attendance, allergy, pacifier use and exposure to tobacco smoke, as reviewed by Daly *et al.* [4]. Therefore, we assessed several covariates that could influence OM with results presented in Table 3. Attendance at day care was the most significant environmental covariate associated with OM risk in this cohort (day care in 1^st^ year OR = 2.23; P = 4.86×10^−8^; day care ever in yrs1–3 OR = 1.76; P = 1.03×10^−5^) followed by diagnosis of allergy (in 3^rd^ year OR = 1.94; P = 3.14×10^−6^; allergy diagnosis ever in yrs1–3 OR = 1.47; P = 0.0005) and non-exclusive breastfeeding (i.e. being introduced to other milks or supplements) before the age of 6 months (OR = 1.23; P = 0.03).

**Table 3:** Odds ratios (OR) and P values for demographic, environmental and clinical covariates potentially influencing OM susceptibility in childhood in the Raine Study cohort.

Covariate	% Controls	% Cases	OR	P value	CI
Gender (% Male)	49.8	53.9	1.17	0.07	0.99–1.40
Breast feeding duration (% <6 months)	47.8	49.2	1.06	0.55	0.88–1.27
Other milk introduced (% <6 months)	60.4	65.2	1.23	**0.03**	1.01–1.49
Other milk introduced (% <4 months)	47.7	50.7	1.13	0.20	0.94–1.36
Gestation <37 weeks	11.1	10.7	0.96	0.77	0.72–1.27
Low Birth Weight (<2500 g)	9.2	7.5	0.80	0.16	0.57–1.10
Pet exposure ever (years 1–3)	80.6	81.1	1.03	0.80	0.81–1.31
Reflux (i.e. vomit feeds)	30.0	32.6	1.12	0.23	0.93–1.38
Serious Reflux (i.e. medicated, GP visit)	9.9	10.8	1.10	0.53	0.81–1.49
Attended day care ever in year 1–3	67.2	78.2	1.74	**1.03×10^−5^**	1.36–2.26
Attended day care in year 1	11.3	22.1	2.23	**4.86×10^−8^**	1.68–2.96
Attended day care in year 2	18.8	31.7	1.99	**9.61×10^−8^**	1.56–2.57
Attended day care in year 3	51.2	63.9	1.68	**8.77×10^−7^**	1.36–2.08
Asthma diagnosed ever	45.6	48.1	1.10	0.34	0.91–1.35
Smoke exposure ever (years 1–3)	44.7	41.5	0.88	0.20	0.72–1.07
Allergy ever (years 1–3)	29.0	37.5	1.47	**0.0005**	1.18–1.81
Allergy in year 1	9.6	14.2	1.55	**0.002**	1.18–2.05
Allergy in year 2	11.6	14.8	1.33	0.06	0.99–1.78
Allergy in year 3	9.3	16.6	1.94	**3.14×10^−6^**	1.48–2.55
Income yr 1 (Below av*; <$24,000AUD)	41.5	38.7	0.89	0.24	0.74–1.08
Income yr 2 (Below av*; <$27,000AUD)	46.8	42.7	0.85	0.11	0.69–1.04
Income yr 3 (Below av*; <$27,000AUD)	43.1	40.1	0.89	0.22	0.73–1.07
Maternal education ≤ year 12	26.3	26.4	1.00	0.95	0.80–1.26
Maternal education > year 12	19.3	23.2	1.27	0.06	0.99–1.61

Covariate data collected as part of the Raine Study follow-up in years 1–3. P values and 95% confidence intervals (CI) determined via logistic regression. * - below average income for years 1 to 3 determined via the Australian Bureau of Statistics (http://www.abs.gov.au/) for the year in question. Significant P values are highlighted in bold.

### Influence of environmental and clinical covariates on genetic associations

To determine whether environmental or clinical covariates influenced genetic associations we examined data for a subset of 831 (256 cases; 575 controls) genotyped Raine Study participants for whom full covariate data were available. Genome-wide analysis adjusting for PCs in this subset of participants highlighted several other genes/regions (Table 4) containing multiple significantly associated SNPs (P_adj-PCA_<10^−5^). This includes the *GALNT14* (located ∼34 kb distal of *CAPN14* on 2p23.1; best P_adj-PCA_ = 2.2×10^−5^), *ASPH* (8q12.3; best P_adj-PCA_ = 3.85×10^−6^), *ALDH1A2* (15q21.3; best P_adj-PCA_ = 3.72×10^−5^), and *RPTOR* (17q25.3; best P_adj-PCA_ = 4.92×10^−5^) genes. Estimates of the *β* coefficient for each SNP at these genes, and in the top genes of interest (*CALPN14*, *BPIFA1*, *BPIFA3*, *BPIFA4P*) derived from the full discovery GWAS and VEGAS analyses, did not change appreciably after adjustment for covariates (Table S1) in this dataset, indicating that genetic associations were independent of these covariates.

**Table 4:** Top 25 genotyped polymorphisms, adjusted for the first two PCs, ordered by chromosome and physical location, from the GWAS analysis for the subset of Raine Study participants for whom full covariate data (day care attendance, allergy and non-exclusive breast feeding) were available.

SNP	Chr	Physical Location	Major/Minor	MAF	Risk Allele	Gene/Region^a^	P_adj-PCA_	OR_adj-PCA_(95% CI)
rs2098787	2	31150148	G/A	0.470	G	GALNT14	3.17×10^−5^	1.58 (1.28–1.97)
rs1862981	2	31151028	C/A	0.463	C	GALNT14	2.20×10^−5^	1.60 (1.29–1.99)
rs2377445	2	106027133	G/A	0.303	G	INTERGENIC	5.20×10^−5^	1.57 (1.26–1.96)
rs17624623	3	61595506	A/G	0.410	A	PTPRG	2.02×10^−5^	1.60 (1.29–1.98)
rs10008015	4	106224696	A/G	0.113	A	RP11-556I14.1	9.32×10^−6^	2.08 (1.50–2.87)
rs1859161	4	106262141	A/G	0.090	A	RP11-556I14.1	2.57×10^−6^	2.32 (1.63–3.29)
rs6826919	4	113023321	G/A	0.428	A	RP11-269F21.1	4.05×10^−5^	0.63 (0.51–0.79)
rs1457955	6	77468785	C/A	0.133	C	INTERGENIC	4.98×10^−5^	1.85 (1.37–2.49)
rs4709819	6	164383345	G/A	0.395	A	INTERGENIC	2.67×10^−6^	0.63 (0.50–0.78)
rs10242197	7	90097848	G/A	0.193	A	CDK14	4.84×10^−5^	0.54 (0.41–0.73)
rs2882460	8	62688450	C/A	0.208	C	ASPH	4.96×10^−5^	1.66 (1.30–2.12)
rs6471969	8	62729386	C/A	0.184	C	ASPH	3.85×10^−6^	1.83 (1.42–2.37)
rs11990408	8	62747549	A/G	0.192	A	ASPH	1.10×10^−5^	1.76 (1.27–2.27)
rs11787089	8	62783393	G/A	0.150	G	ASPH	6.83×10^−6^	1.88 (1.43–2.47)
rs16919668	10	20319164	A/G	0.163	G	PLXDC2b	3.28×10^−5^	0.50 (0.36–0.70)
rs1336708	13	101763004	A/G	0.225	G	FGF14	5.17×10^−5^	0.57 (0.44–0.75)
rs4512966	13	109880059	G/A	0.500	G	COL4A2	5.01×10^−5^	1.55 (1.25–1.91)
rs6493973	15	56079928	G/A	0.029	G	ALDH1A2	4.40×10^−5^	3.43 (1.90–6.20)
rs2218261	15	56081314	G/A	0.029	G	ALDH1A2	4.40×10^−5^	3.43 (1.90–6.20)
rs2704219	15	56115740	A/G	0.031	A	ALDH1A2	3.72×10^−5^	3.39 (1.90–6.05)
rs11658127	17	64225877	A/G	0.127	A	INTERGENIC	2.29×10^−5^	1.93 (1.42–2.61)
rs11658297	17	64240578	G/A	0.126	G	INTERGENIC	1.89×10^−5^	1.94 (1.43–2.63)
rs4627412	17	76138839	A/G	0.307	A	RPTOR	4.92×10^−5^	1.58 (1.27–1.96)
rs9911978	17	76139002	A/G	0.307	A	RPTOR	4.92×10^−5^	1.58 (1.27–1.96)
rs2839520	21	42742732	A/G	0.464	G	UBASH3A	1.63×10^−5^	0.63 (0.51–0.77)

Where MAF  =  minor allele frequency, 95% CI = 95% confidence intervals. **^a^ -** Gene/Region is based on annotation from Ensembl build 54; intergenic SNPs fall >20 kb from an annotated gene.

b **-** Odds ratios are shown for the major allele.

Data for GWAS analyses adjusted for these clinical and environmental covariates are presented in Table S1.

**Figure 3:**
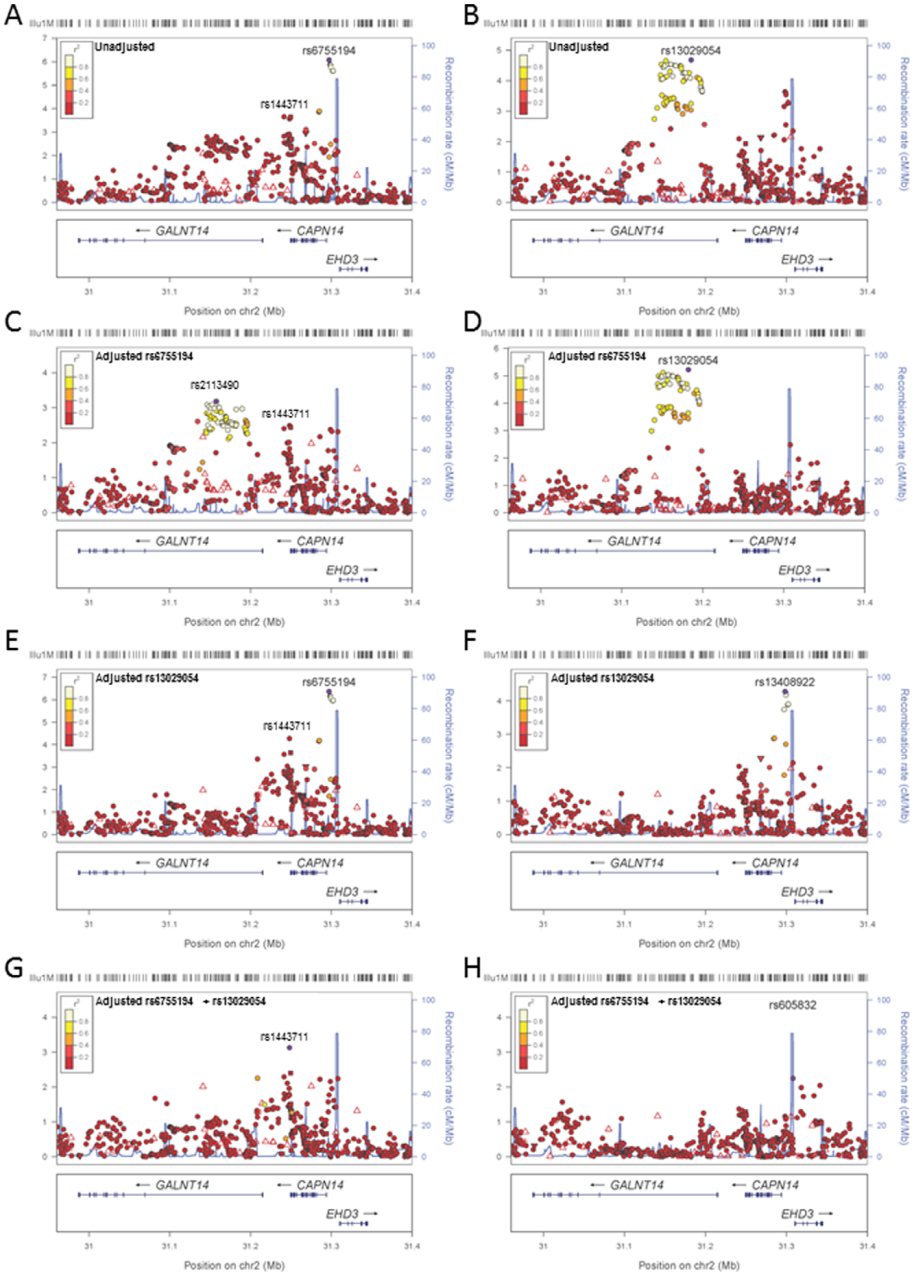
Regional LocusZoom plots of association for the *CAPN14* and *GALNT14* genes. LocusZoom plots of association in the imputed dataset adjusted for PCs across the *CAPN14-GALNT14* gene region in (A) the full discovery set of 1491 Raine Study participants and (B) the subset of 831 Raine Study participants for whom full covariate data were available. (C) and (D) present results in these two study groups, respectively, after conditioning on the top SNP rs6755194 at *CAPN14*; (E) and (F) after conditioning on the top SNP rs13029054 at *GALNT14*; and (G) and (H) after conditioning on both of these SNPs. Top associated SNPs in each plot are represented by purple circles, the colour of all other SNPs is representative of the pairwise r^2^ value with the relevant top SNP using patterns of LD from the Raine Study cohort. Recombination rates are shown by the solid blue line. Physical positions are based on NCBI Build 36 of the human genome.

This analysis further highlighted the region of chromosome 2p23.1 as of particular interest in containing both *GALNT14* encoding N-acetylgalactosaminyltransferase 14 and *CAPN14*. Figure 3A plots the imputed dataset across this region from the full discovery GWAS analysis, highlighting the peak of association at *CAPN14*. The imputed analysis identified 7 additional strongly associated SNPs (8.3×10^−7^<P_adj-PCA_<2.5×10^−6^) located 3.25–8.32 kb upstream of *CAPN14* (Figure 3A) at chromosome 2p23.1. Figure 3B plots the imputed dataset across this region in the subset of Raine participants for whom full covariate data were available, highlighting the peak of association at *GALNT14*. This imputed analysis supports association at *GALNT14* with a total of 31 SNPs (2.65^−5^<P_adj-PCA_<9.86×10^−5^) associated at P_adj-PCA_<10^−4^ (Figure 3B). In both the full discovery sample analysis (Figure 3C) and the Raine dataset with full covariates (Figure 3D), adjusting for the top *CAPN14* SNP rs6755194 reduced the signal at *CAPN14* but maintained (Figure 3C) or improved (Figure 3D) the signal at *GALNT14*. Conversely, adjusting for the top *GALNT14* SNP rs13029054 reduced the signal at *GALNT14* but maintained (Figure 3E) or improved (Figure 3F) the signal at *CAPN14*. Logistic regression modelling confirmed that addition of SNP rs13029054 compared to a model in which rs6755104 was considered alone added significant independent effects (P_LRT_ = 0.0027 for the full discovery GWAS dataset; P_LRT_ = 4.25×10^−6^ for the covariate dataset). Conversely, addition of rs6755104 compared to a model in which rs13029054 was considered alone added significant independent effects (P_LRT_ = 5.67×10^−7^ for the full discovery GWAS dataset; P_LRT_ = 1.98×10-4 for the covariate dataset). Independent association signals at *CAPN14* and *GALNT14* is consistent with analysis showing that there is no long range LD between these two genes (pairwise D′≤0.20; r^2^ = 0). The presence of independent association signals *CAPN14* (P_Gene_ = 5.3×10^−5^) and *GALNT14* (P_Gene_ = 2.4×10^−3^) is also supported by the gene-based analysis in VEGAS in the full discovery GWAS sample. Adjusting for both SNPs (Figures 3G and 3H) reduced the signal across *GALNT14*, but retained a weaker signal (Figure 3G; P_adj-PCA_ = 7.34×10^−4^ at rs1443711), suggesting that there may be more than one association signal at *CAPN14*. This second signal was also retained in the full discovery cohort following adjustment for the top SNPs at *CAPN14* SNP rs 6755194 (Figure 3C).

### Interrogating the GWAS data for replication of published associations for OM

A number of genes have previously been implicated in the pathogenesis of OM, through both murine models and human candidate gene studies. To determine whether any of these previously implicated genes show evidence of association in the Raine Study, we performed a directed interrogation of the discovery GWAS data for all SNPs across each of the previously associated genes (see Table S2). Human candidate gene studies have previously identified 20 genes as showing significant association with OM susceptibility (Table S2A) [11]. Evidence for association (nominal P≤0.05, i.e. without correction for multiple testing) is observed at several of these genes, including *IL10* (P_adj-PCA_ = 0.010), *TNF* (P_adj-PCA_ = 0.035), *SFTPD* (P_adj-PCA_ = 0.033) and *MUC2* (P_adj-PCA_ = 0.003), none of which achieved the required threshold (P = 0.002; 0.05/20) to take account of multiple testing of 20 genes included in this candidate gene hypothesis. Murine models of OM have also identified several OM genes [32]. In addition to our previously published data on *FBXO11* [13], investigation of the human homologues of 25 of these genes (Table S2B) provided evidence of association (nominal P≤0.05) at four additional genes, including TP73 (P_adj-PCA_ = 0.011), *DNAH5* (P_adj-PCA_ = 0.015), *DNAH11* (P_adj-PCA_ = 0.016) and *FAS* (P_adj-PCA_ = 0.021), with only *FBXO11* (P_adj-PCA_ = 4.64×10^−5^) achieving a P-value that would meet the threshold required to allow for multiple testing of 25 genes (P = 0.002; 0.05/25) to test this mouse-to-man hypothesis.

### Interrogating the GWAS data for replication of published linkages for OM

A number of regions of linkage (chromosomes 3p25.3, 10q22.3, 10q26.3, 17q12, 19q13.4) have also been reported following genome-wide analysis of microsatellite panels in family studies of otitis media [8], [9], [10]. We looked across these regions of linkage (Figure S2) for evidence provided by top SNPs (Table S2C). VEGAS support for gene-based associations were observed at *SRGAP3* (P_Gene_ = 0.044) but not *ATP2B2* (P_Gene_ = 0.069) at 3p25.3 (Figure S2A), at *TCERG1L* (P_Gene_ = 0.012) and *PP2R2D* (P_Gene_  = 0.007) but not *GLRX3* (P_Gene_ = 0.5) at 10q26.3 (Figure S2B), and at *CCT6B* (P_Gene_ = 0.026) and *ZNF830* (P_Gene_ = 0.022) but not *SLFN5* (P_Gene_ = 0.3) at 17q12 (Figures S2C and S2D). There was no VEGAS gene-based support for association at *LILRA2* (P_Gene_ = 0.1) which contained the best SNP-hit across the region (Table S2C), or any other genes in the region of linkage at 19q13.4 (data not shown).

### Replication analysis of GWAS candidates

As no single variant achieved genome-wide significance in the Raine GWAS analyses, and many regions were observed with SNPs at P_adj-PCA_<10^−5^ (32 genomic regions), we carried out Ingenuity Pathway Analysis (Ingenuity® Systems) seeded with top genes from the discovery GWAS to identify potentially interesting candidate genes for replication in the WAFSOM. Given our published data showing replication at *FBXO11* across the Raine Study and WAFSOM cohorts [13], and the evidence of others for activation of the transforming growth factor beta (TGFβ) pathway in OM [34], we focused initially on a number of genes that interact within this pathway (Figure S3), including the *GALNT14* gene. In total seven candidate genes were selected for replication analysis, including the top hits from the PC and fully adjusted analyses plus 5 additional genes identified via pathway analysis. Specifically we genotyped 20 tag-SNPs (Table S3) within the *CAPN14*, *GALNT14*, *GALNT13*, *BMP5*, *NELL1*, *TGFB3* and *BPIFA1* genes in 645 families, containing 793 affected individuals (55% rAOM; 45% COME), from the WAFSOM. No SNPs were found to significantly deviate from HWE.

Results from family based robust case/pseudo-control conditional logistic regression analysis under an additive model showed that none of the 20 tag-SNPs included in the replication analysis was associated (nominal P_robust_<0.05) with OM in the WAFSOM cohort (Table S4). This was confirmed using FBAT (data not shown). Stratified analysis (data not shown) using only families that self-reported as Caucasian (601 families), or restriction of analysis to just those children diagnosed with rAOM only (N = 190) or COME only (N = 57), did not reveal any other significant associations that withstood correction for multiple testing, although the latter were underpowered. Analysis of LD patterns between replication SNPs in the Raine Study and WAFSOM cohorts show that patterns of LD are comparable between the two cohorts (Figure S4).

## Discussion

Here we report the results from the first GWAS of OM carried out using data from 1,491 participants in the Raine Study. Although we cannot be certain that associations observed were not due to type I errors, a number of SNPs located at or close to novel candidate genes were identified that may have a putative role in OM susceptibility. This includes the *CAPN14* and *GALNT14* genes at 2p23.1, and the *BPIFA3* and *BPIFA1* at 20q11.21, none of which has previously been implicated in OM pathogenesis. Other than *FBXO11* [12], [13], no SNPs within regions identified in previous candidate gene or genome-wide linkage studies showed association at P<10^−5^. Pathway analysis was used to identify additional genes that cluster within relevant functional pathways related to the data from the discovery GWAS presented here. This revealed that several genes from the discovery GWAS are either members of, or interact with, the TGFβ pathway.

To replicate the results from this discovery GWAS we undertook additional genotyping of variants in seven genes, including the *CAPN14* and *GALNT14* genes plus five additional genes from the TGFβ pathway (*BMP5, GALNT13, NELL1*, *TGFB3* and *BPIFA1*), in families that were available as part of the larger WAFSOM cohort. The results of this replication analysis did not provide evidence for association with OM at any of the seven genes.

The principle factors that may have contributed to the lack of replication between cohorts used in this study are sample size and phenotypic heterogeneity. For example, the sample of 416 Raine Study cases and 1,075 controls was only 54.6% powered to detect associations at an alpha level of P = 1×10^-5^ with effect size (OR = 1.5) for SNPs with MAF>0.1. For most common, complex diseases where small genetic effect sizes (i.e. OR<1.5) are expected, a sample size in excess of 1000 cases and 1000 controls is recommended [35]. In addition, there was phenotypic heterogeneity as there was incomplete overlap in phenotypes across the OM spectrum in the Raine discovery and WAFSOM replication cohorts indicating that the WAFSOM cohort may not represent a true replication cohort. The phenotype in the Raine Study is based on a once yearly clinical examination or parental reporting of ≥3 AOM episodes during the first three years of life, with 65% of individuals documented with AOM, MEE, a scarred tympanum, or tympanostomy tubes at the time of a clinical examination with/without parent report of >3 episodes of OM per year, and 35% based on the parent-reported phenotype alone. This phenotype is therefore biased towards the milder end of the OM spectrum compared to the WAFSOM study, where all probands and their affected siblings were referred to an ENT specialist who had either undertaken or recommended tympanostomy tube insertion for the treatment of rAOM (i.e. ≥3 episodes in 6 months or ≥4 episodes in 12 months; 55% of cases) or COME (i.e. MEE ≥3 months; 45% of cases). Thus, the WAFSOM cohort includes only the severe end of the OM spectrum. Our results might also reflect “winner's curse”, which predicts that genetic effect sizes reported from discovery GWAS are generally inflated, leading to under estimation of replication sample sizes required to replicate observed associations [36]. Whilst the 645 WAFSOM families provided >80% power at an alpha level of 0.0025 (i.e. 0.05/20 SNPs) to detect the genetic effect sizes (i.e. OR≥1.58) observed from the discovery GWAS at both *CAPN14* and *GALNT14* for variants with MAF≤0.10, this may be an under estimation of the replication sample size required. If the true genetic effect sizes were indeed smaller (i.e. OR≤1.4; MAF = 0.10), a replication cohort of at least 1700 families would be required to provide >80% power to detect these genetic effects. In summary, the lack of replication for the genes identified in the Raine discovery GWAS may be explained by factors of sample size and phenotypic heterogeneity, and may replicate in further well-powered cohorts in which these factors are more rigorously controlled.

Despite the lack of replication in WAFSOM and the plausible likelihood that some associations arose due to type I errors, the discovery GWAS analysis in the Raine Study has highlighted genetic risk factors that could play a role in OM, and are of interest in relation to their biological candidacy. For example, CAPN14 is a member of the calpain family of cytosolic calcium-activated cysteine proteases [37], other members of which mediate host cell invasion by known otopathogens [38] and are up-regulated in cholesteatoma associated with OM and its sequelae, including OME, COME and chronic suppurative OM [39]. CAPN14 is up-regulated in human conjunctival epithelial cells by interleukin-4 [40], a key T helper 2 cytokine involved in infection control and allergic responses. GALNT14 belongs to a large subfamily of glycosyltransferases that catalyse the transfer of N-acetyl-D-galactosamine (GalNAc) in O-glycosylation of mucin substrates such as MUC2, MUC5AC, MUC7, and MUC13 [41]. GalNAc containing sugar residues are essential for initial adherence of respiratory bacteria epithelial cell surfaces [42]. As is the case for FBXO11 [43], which regulates anti-inflammatory TGFβ via SMAD2 [44], this could include a role in response to commensal bacteria in determining OM pathogenesis. *BPIFA3* (previously known as *SPLUNC3*) and *BPIFA1* (previously known as *PLUNC* or *SPLUNC1*) are members of the bacterial permeability-increasing (BPI) protein family [45]. Proteins in this family are one of four groups of proteins involved in the early recognition of bacterial pathogens as part of the host defence at the nasopharyngeal, oral and lung entrances [46]. BPIFA1 decreases levels of *Mycoplasma pnuemoniae* and epithelial IL-8 in the airways [47].

In conclusion, we present the results from the first GWAS performed to identify the genetic determinants of OM in childhood. Several genes/pathways of plausible biological relevance are identified and replication of these observations in additional OM cohorts is recommended. This discovery GWAS also highlights factors that should be addressed in future GWAS studies of OM, namely that of phenotypic heterogeneity and sample size. The recent formation of the OTIGEN Consortium (www.otigen.org), an international collaboration between several research groups possessing well-powered, well-phenotyped OM cohorts, should address both of these issues allowing the genetic determinants of OM to be confidently elucidated.

## Supporting Information

Figure S1 **(A) Ethnic spread of Raine Study participants compared to the four HapMap populations.** The Raine Study population (RAINE) is in red, Caucasian population (CEU) in blue, Japanese and Chinese population (JPT+CHB) in green and African population (YRI) in purple. **(B)** Quantile-quantile plot of the observed versus expected log_10_(P-value) following PC adjustment.
(PDF)

Figure S2 **Regional LocusZoom plots of association in the full imputed dataset across three genomic regions previously linked to OM susceptibility: (A) the 3p25.3 region** [**8**] **adjusted for PCs; (B) the 10q26.3 region (linkage peak microsatellite D10S212,** [**8**] **) adjusted for PCs; (C) the 17q12 region** [**9**] **adjusted for PCs, and (D) a smaller region of the 17q12 plot to highlight the gene position for the top SNPs.** The top associated SNPs are represented by purple circles with the colour of all other SNPs representative of the pairwise r^2^ value relative to the top SNP using patterns of LD from the CEU HapMap populations. Recombination rates are shown by the solid blue line. Physical positions and gene designations are based on NCBI Build 36 of the human genome.
(PDF)

Figure S3 **Pathway analysis using Ingenuity Pathway Analysis reveals many genes harbouring associated SNPs from the discovery GWAS (highlighted in gray) are part of, or interact with, the TGFβ pathway.**
(PDF)

Figure S4 **Comparison of LD patterns in replicated genes between the Raine Study and WAFSOM cohorts.**
(PDF)

Table S1 **Analysis in GenABEL showing the influence of adjustment for clinical and environmental covariates on associations with OM in 831 Raine Study participants for whom full covariate data were available.** Data are shown for PCA adjustment only (P_adj-PCA_), and with additional adjustment for covariates including daycare attendance <3 yrs (P_adj-daycare_), allergy at < 3 yrs (P_adj-allergy_), non-exclusive breast feeding at <6 mths (P_adj-supp_), and all covariates (P_adj-allCov_).
(PDF)

Table S2 **Directed interrogation of the Raine Study Discovery GWAS results across genes/regions previously implicated in childhood OM susceptibility in the literature: (A) candidate genes previously reported to be associated with OM susceptibility; (B) the human homologues of genes identified from murine models of OM; and (C) regions of the human genome previously implicated in genome-wide linkage studies.** For all genes/regions and SNPs the physical location is based on annotation from Ensembl release 54 with gene boundaries defined as +/- 20 kb from the 5′ and 3′ UTR. Results are presented from the discovery GWAS adjusted for PCs alone. This analysis was undertaken to find any evidence of association at these genes, not as a direct replication of specific SNPs reported in other studies. Nominal P<0.05 are highlighted in bold. No associations were robust to correction for the number of genes interrogated under each hypothesis (see main text).
(PDF)

Table S3 **SNPs/Genes selected for replication analysis in the WAFSOM cohort.**
(PDF)

Table S4 **Replication of selected genes in the WAFSOM cohort using case/pseudo-control conditional logistic regression under an additive model.** Where possible, tag-SNPs were selected to match SNPs genotyped in the Raine cohort. For some genes we selected additional tag-SNPs to cover areas of the gene not well covered on the SNP-chip used to analyse Raine. P_adj-PCA_ values from the Raine cohort are provided for ease of comparison.
(PDF)
