# Supplementary material for: Genome-Wide Association Study to Identify the Genetic Determinants of Otitis Media Susceptibility in Childhood
Source: PLoS One. 2012 Oct 25;7(10):e48215. doi: 10.1371/journal.pone.0048215 (PMC3485007; doi:10.1371/journal.pone.0048215)

Figure S1. (A) Ethnic spread of Raine Study participants compared to the four HapMap populations. The Raine Study population (RAINE) is in red, Caucasian population (CEU) in blue, Japanese and Chinese population (JPT+CHB) in green and African population (YRI) in purple. **(B)** Quantile-quantile plot of the observed versus expected $\log_{10}$(P-value) following PC adjustment.

(A)

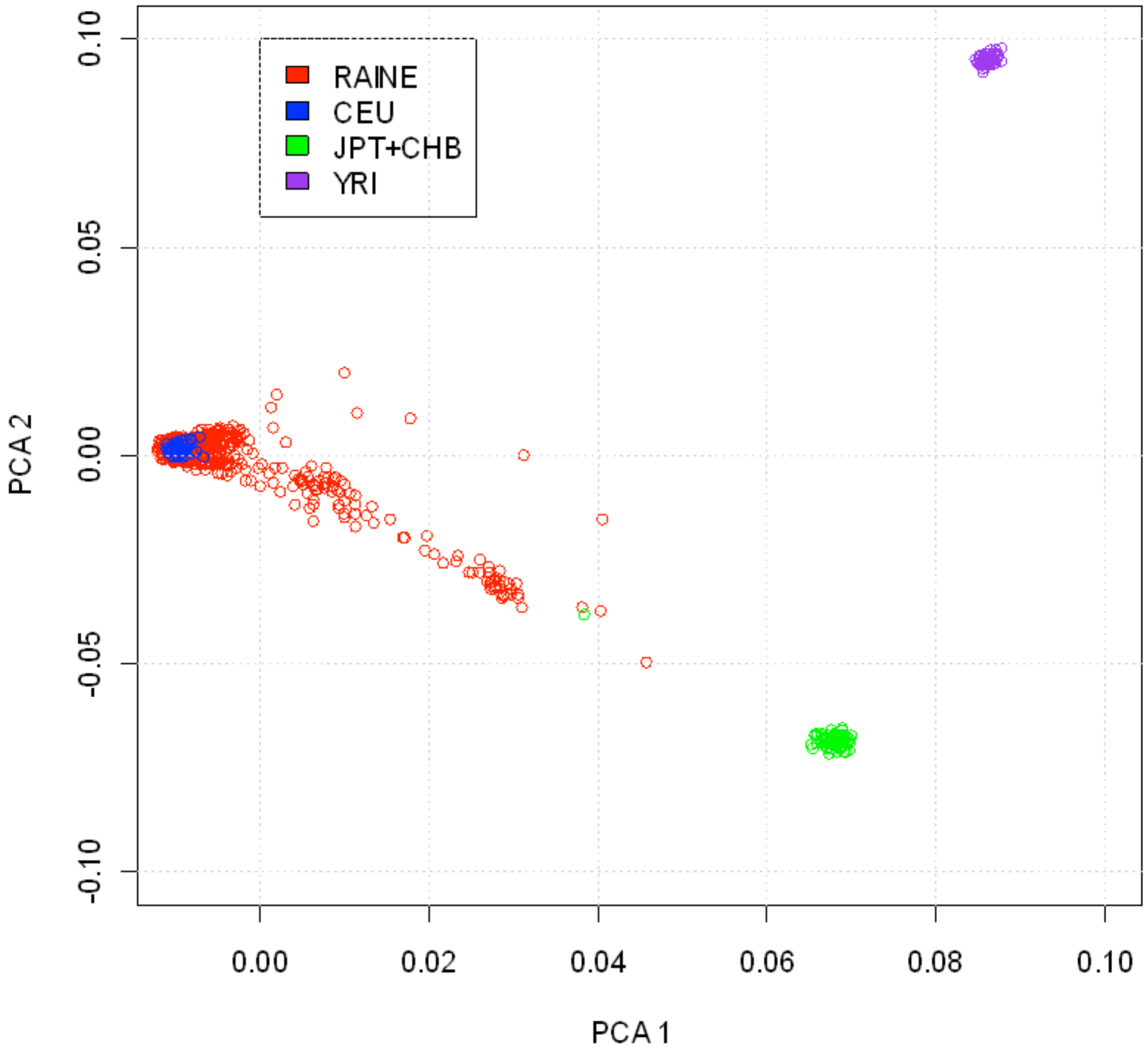

(B)

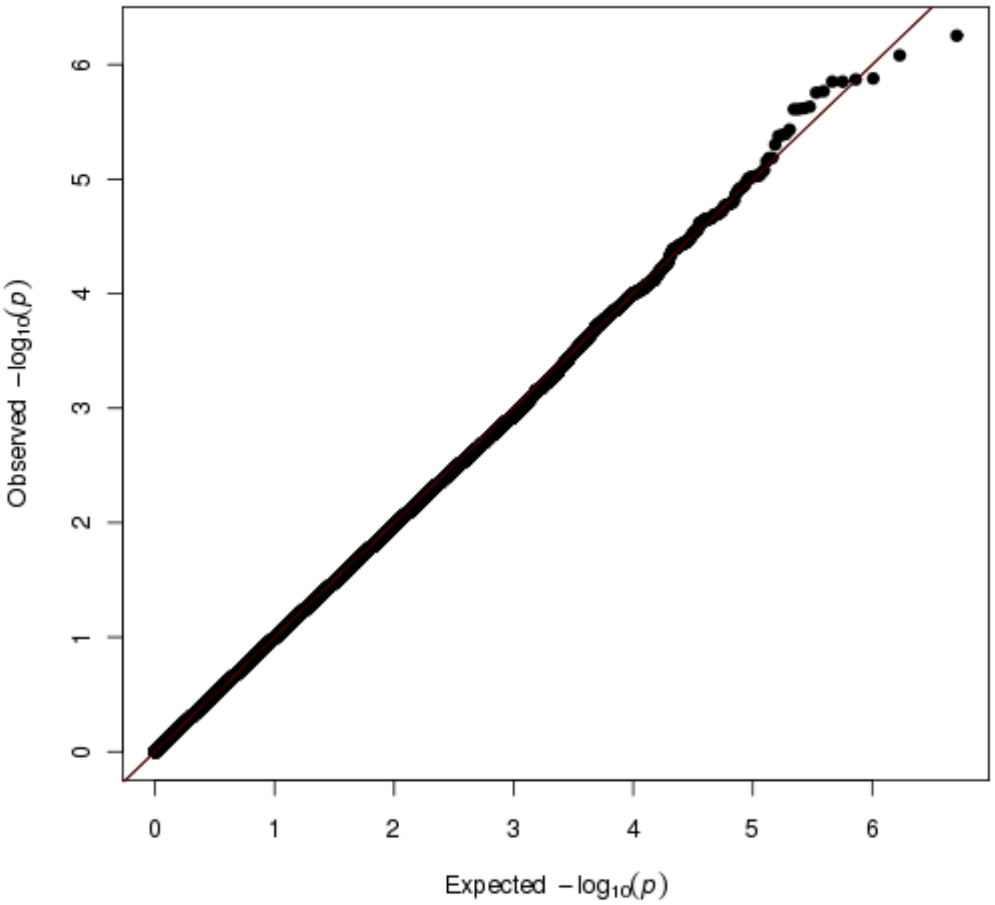

Supplement: Figure S1 — (A) Ethnic spread of Raine Study participants compared to the four HapMap populations. The Raine Study population (RAINE) is in red, Caucasian population (CEU) in blue, Japanese and Chinese population (JPT+CHB) in green and African population (YRI) in purple. (B) Quantile-quantile plot of the observed versus expected log10(P-value) following PC adjustment. (PDF) [file pone.0048215.s001.pdf]
