# Supplementary material for: Genome-Wide Association Study to Identify the Genetic Determinants of Otitis Media Susceptibility in Childhood
Source: PLoS One. 2012 Oct 25;7(10):e48215. doi: 10.1371/journal.pone.0048215 (PMC3485007; doi:10.1371/journal.pone.0048215)
Supplement: Figure S2 — Regional LocusZoom plots of association in the full imputed dataset across three genomic regions previously linked to OM susceptibility: (A) the 3p25.3 region [8] adjusted for PCs; (B) the 10q26.3 region (linkage peak microsatellite D10S212, [8] ) adjusted for PCs; (C) the 17q12 region [9] adjusted for PCs, and (D) a smaller region of the 17q12 plot to highlight the gene position for the top SNPs. The top associated SNPs are represented by purple circles with the colour of all other SNPs representative of the pairwise r2 value relative to the top SNP using patterns of LD from the CEU HapMap populations. Recombination rates are shown by the solid blue line. Physical positions and gene designations are based on NCBI Build 36 of the human genome. (PDF) [file pone.0048215.s002.pdf]

Figure S2: Regional LocusZoom plots of association in the full imputed dataset across three genomic regions previously linked to OM susceptibility: (A) the 3p25.3 region [8] adjusted for PCs; (B) the 10q26.3 region (linkage peak microsatellite D10S212, [8]) adjusted for PCs; (C) the 17q12 region [9] adjusted for PCs, and (D) a smaller region of the 17q12 plot to highlight the gene position for the top SNPs. The top associated SNPs are represented by purple circles with the colour of all other SNPs representative of the pairwise r^2 value relative to the top SNP using patterns of LD from the CEU HapMap populations. Recombination rates are shown by the solid blue line. Physical positions and gene designations are based on NCBI Build 36 of the human genome.

(A)

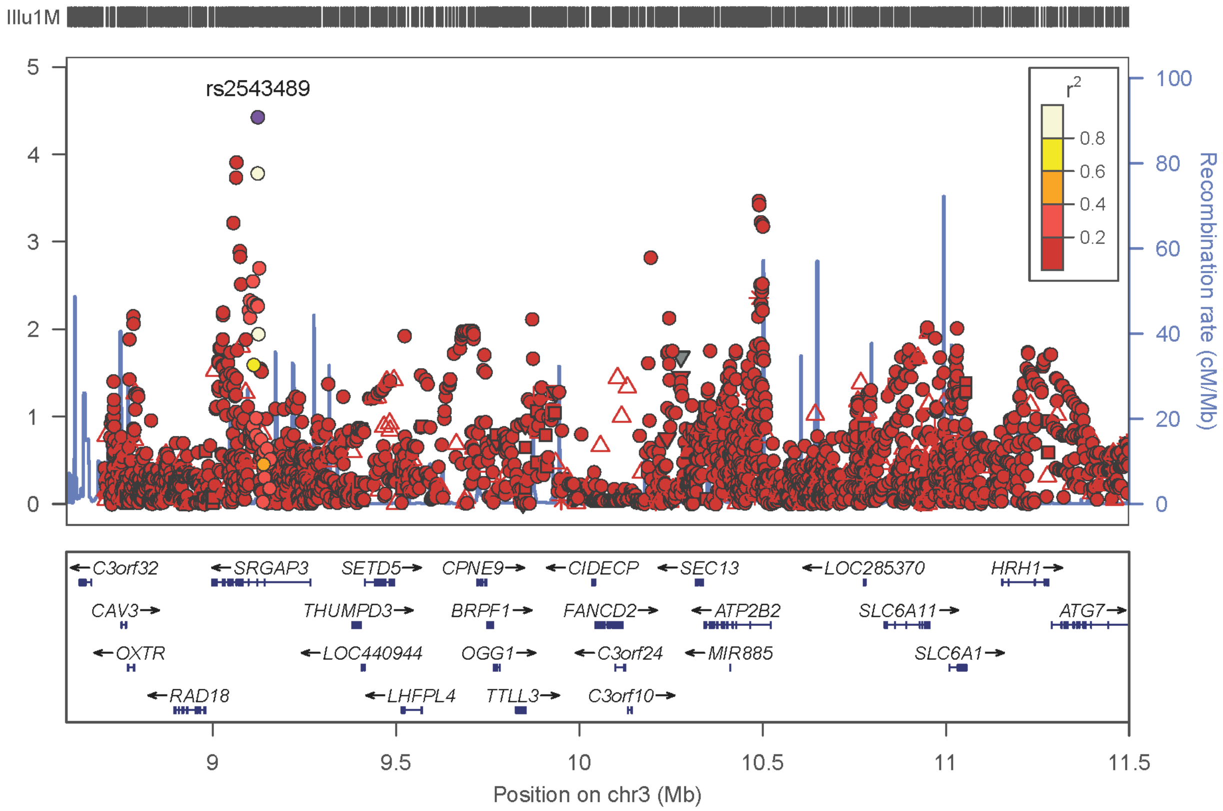

(B)

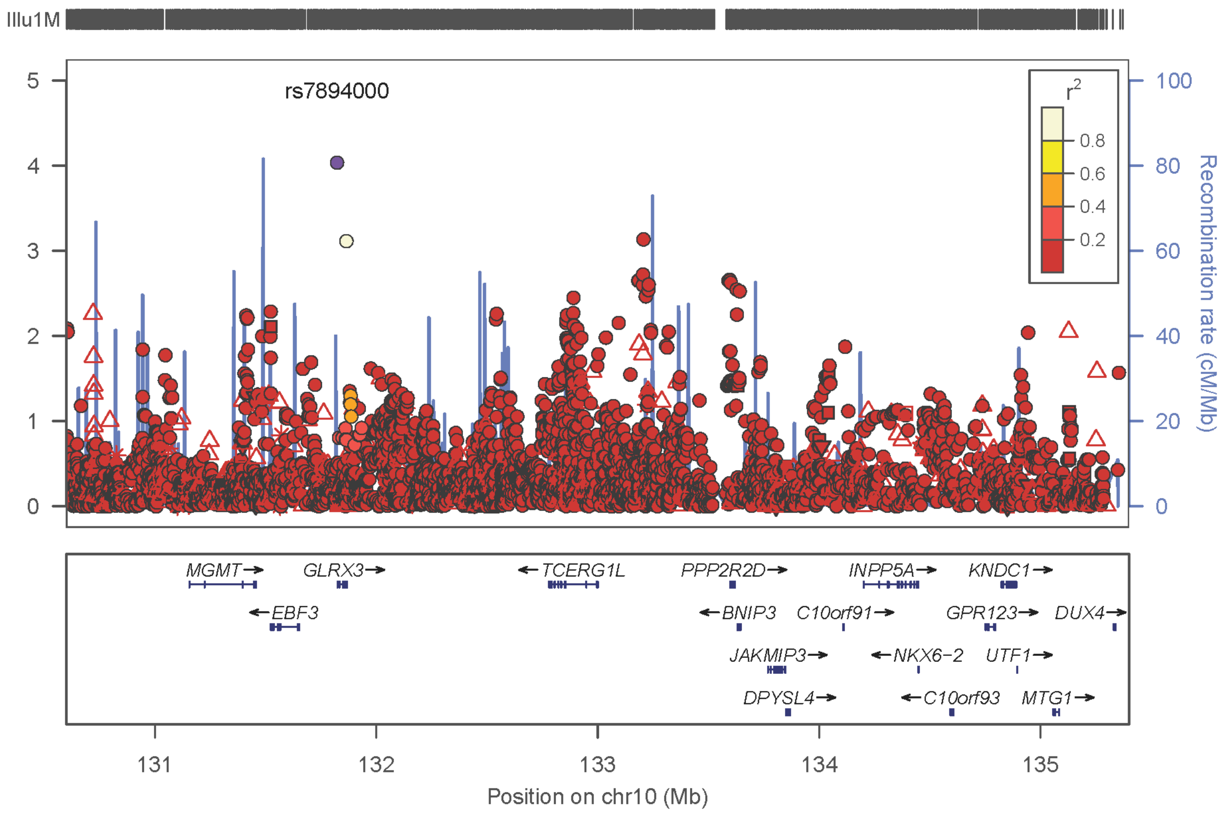
Illu1M
rs7894000
r²
0.8
0.6
0.4
0.2
Recombination rate (cM/Mb)
0
1
2
3
4
5
0
20
40
60
80
100
MGMT
GLRX3
TCERG1L
PPP2R2D
INPP5A
KNDC1
EBF3
BNIP3
C10orf91
GPR123
DUX4
JAKMIP3
NKX6-2
UTF1
DPYSL4
C10orf93
MTG1
131
132
133
134
135
Position on chr10 (Mb)

(C)

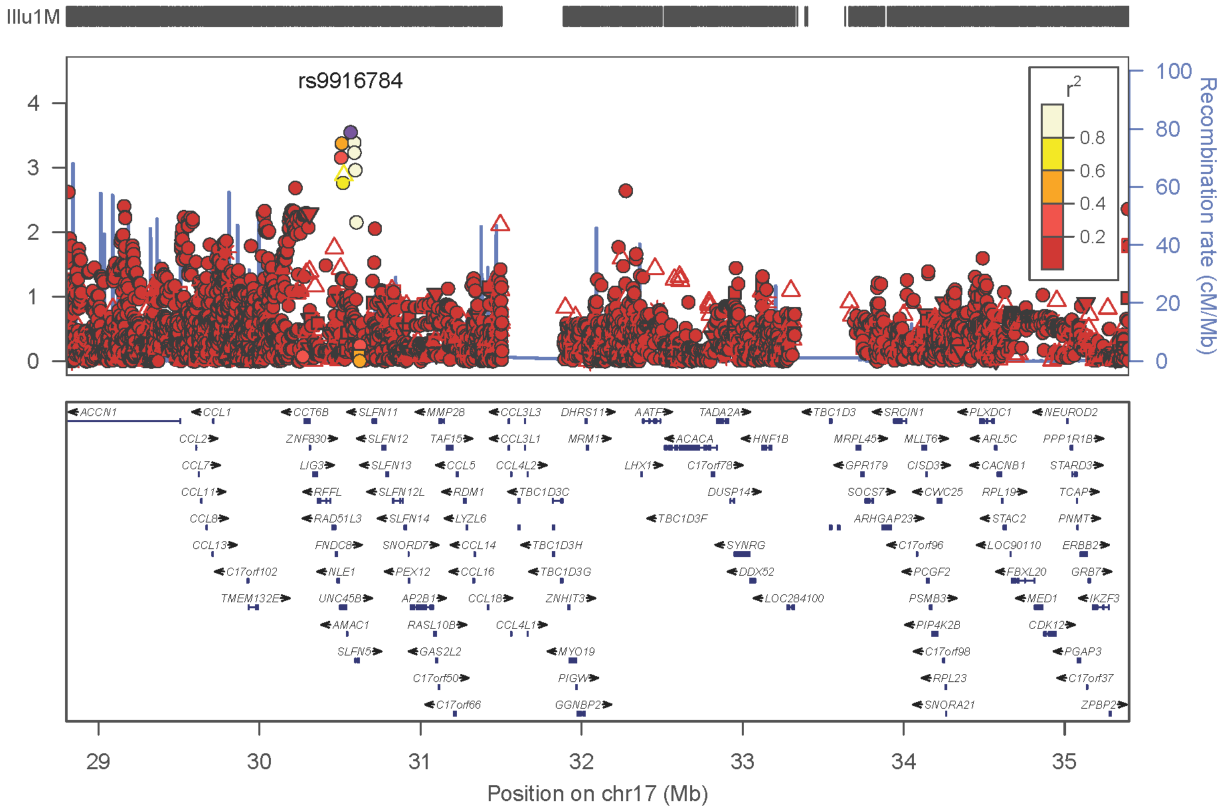
Illu1M
rs9916784
r²
0.8
0.6
0.4
0.2
Recombination rate (cM/Mb)
0
1
2
3
4
0
20
40
60
80
100
ACCN1
CCL1
CCT6B
SLFN11
MMP28
CCL3L3
DHRS11
AATF
TADA2A
TBC1D3
SRCIN1
PLXDC1
NEUROD2
CCL2
ZNF830
SLFN12
TAF15
CCL3L1
MRM1
ACACA
HNF1B
MRPL45
MLLT6
ARL5C
PPP1R1B
CCL7
LIG3
SLFN13
CCL5
CCL4L2
LHX1
C17orf78
GPR179
CISD3
CACNB1
STARD3
CCL11
RFFL
SLFN12L
RDM1
TBC1D3C
DUSP14
SOCS7
CWC25
RPL19
TCAP
CCL8
RAD51L3
SLFN14
LYZL6
TBC1D3F
ARHGAP23
STAC2
PNMT
CCL13
FNDC8
SNORD7
CCL14
TBC1D3H
SYNRG
C17orf96
LOC90110
ERBB2
C17orf102
NLE1
PEX12
CCL16
TBC1D3G
DDX52
PCGF2
FBXL20
GRB7
TMEM132E
UNC45B
AP2B1
CCL18
ZNHIT3
LOC284100
PSMB3
MED1
IKZF3
AMAC1
RASL10B
CCL4L1
PIP4K2B
CDK12
SLFN5
GAS2L2
MYO19
C17orf98
PGAP3
C17orf50
PIGW
RPL23
C17orf37
C17orf66
GGNBP2
SNORA21
ZPBP2
29
30
31
32
33
34
35
Position on chr17 (Mb)

(D)

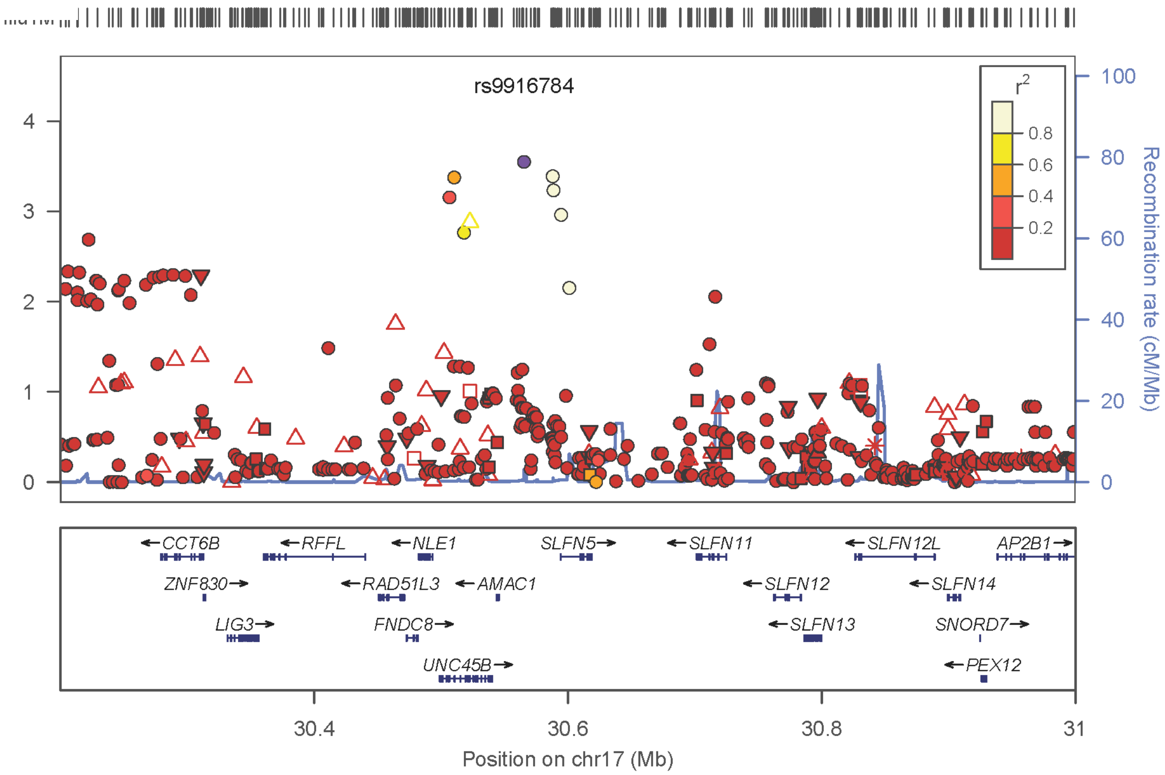
rs9916784
r2
0.8
0.6
0.4
0.2
Recombination rate (cM/Mb)
CCT6B
RFFL
NLE1
SLFN5
SLFN11
SLFN12L
AP2B1
ZNF830
RAD51L3
AMAC1
SLFN12
SLFN14
LIG3
FNDC8
SLFN13
SNORD7
UNC45B
PEX12
Position on chr17 (Mb)
