# Supplementary material for: Genome-Wide Association Study to Identify the Genetic Determinants of Otitis Media Susceptibility in Childhood
Source: PLoS One. 2012 Oct 25;7(10):e48215. doi: 10.1371/journal.pone.0048215 (PMC3485007; doi:10.1371/journal.pone.0048215)

Figure S3. Pathway analysis using Ingenuity Pathway Analysis reveals many genes harbouring associated SNPs from the discovery GWAS (highlighted in gray) are part of, or interact with, the TGFβ pathway.

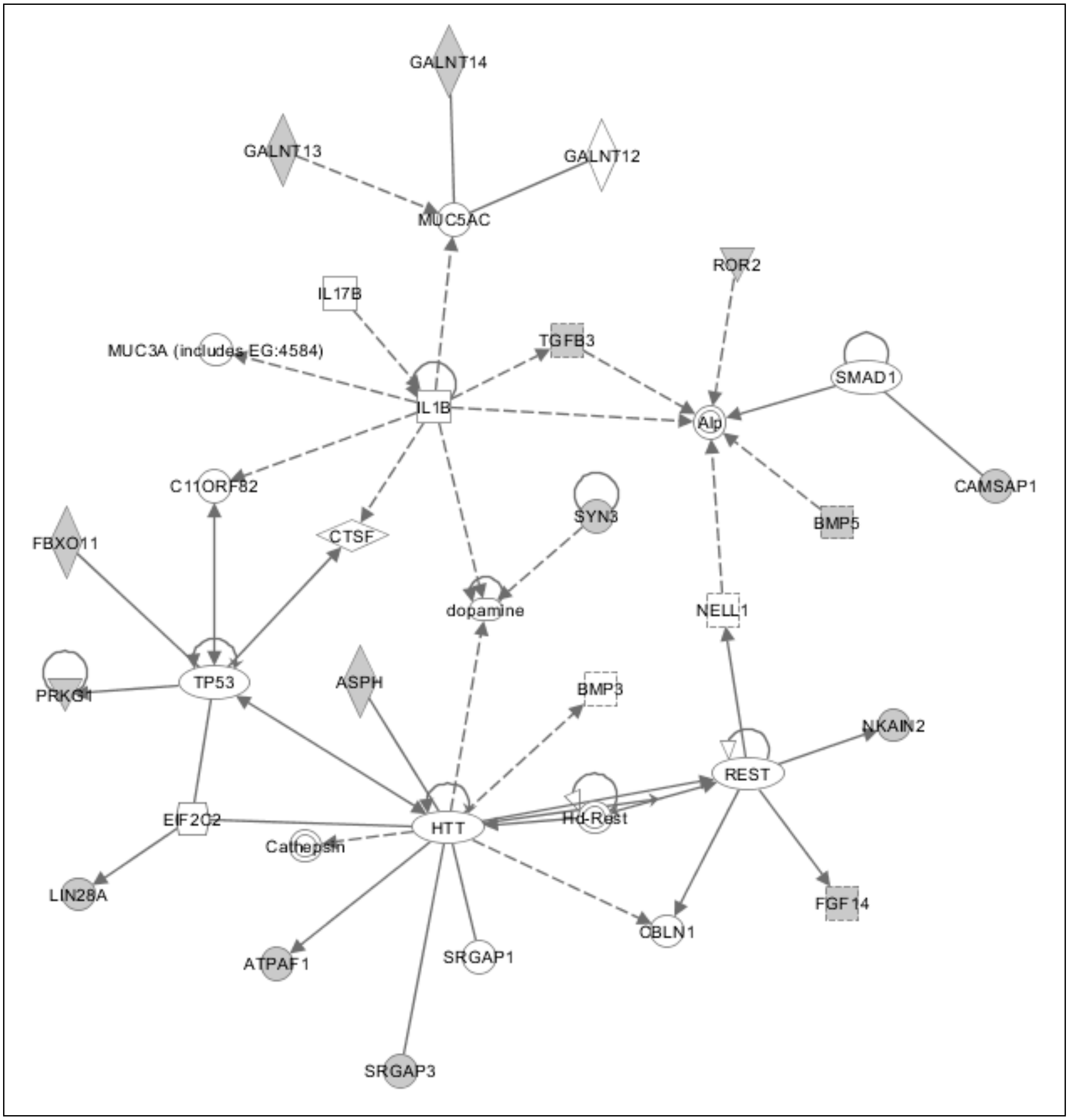

Supplement: Figure S3 — Pathway analysis using Ingenuity Pathway Analysis reveals many genes harbouring associated SNPs from the discovery GWAS (highlighted in gray) are part of, or interact with, the TGFβ pathway. (PDF) [file pone.0048215.s003.pdf]
