# Supplementary material for: Genome-Wide Association Study to Identify the Genetic Determinants of Otitis Media Susceptibility in Childhood
Source: PLoS One. 2012 Oct 25;7(10):e48215. doi: 10.1371/journal.pone.0048215 (PMC3485007; doi:10.1371/journal.pone.0048215)

**Figure S4.** Comparison of LD patterns in replicated genes between the Raine Study and WAFSOM cohorts.

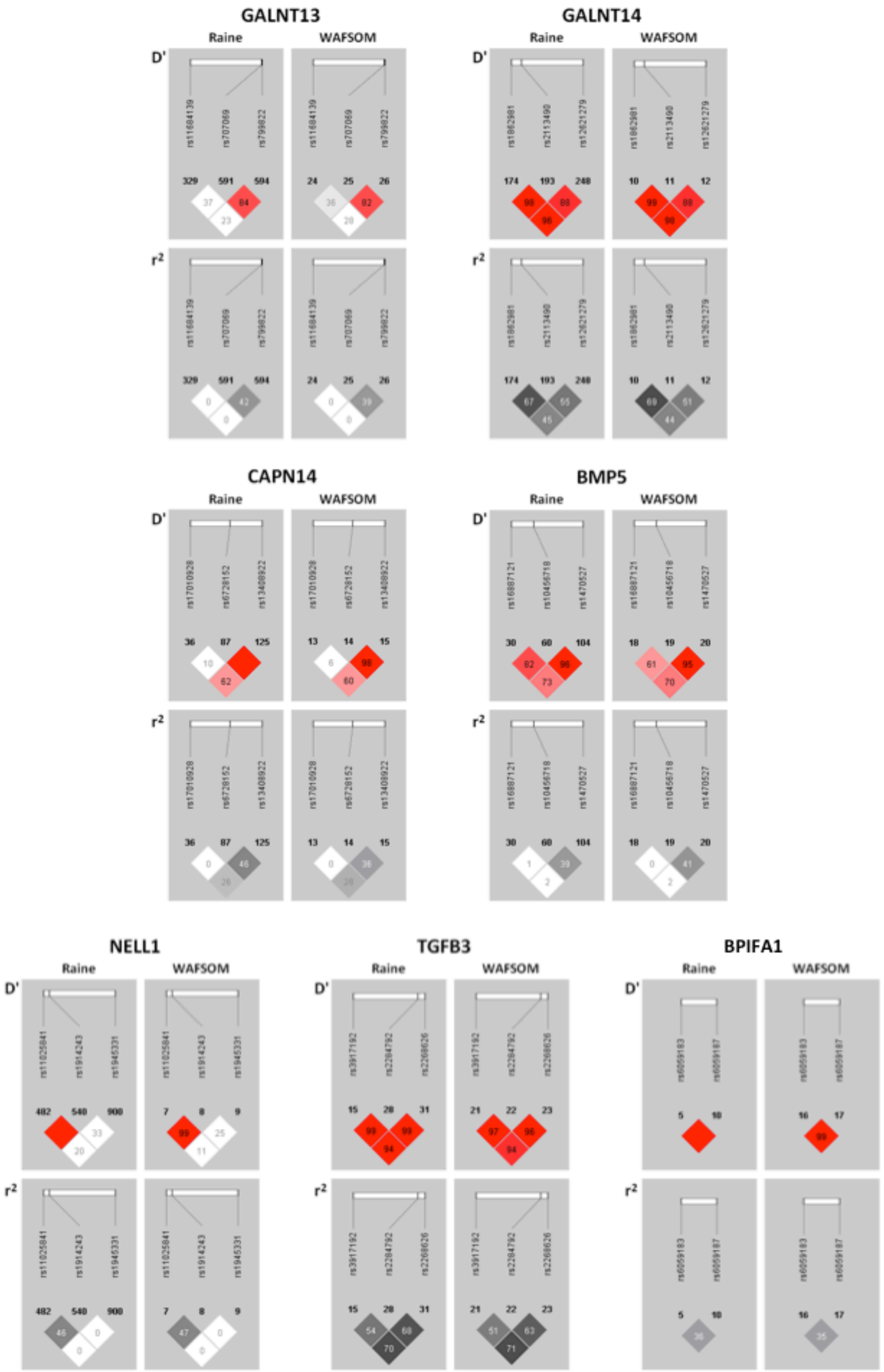

Supplement: Figure S4 — Comparison of LD patterns in replicated genes between the Raine Study and WAFSOM cohorts. (PDF) [file pone.0048215.s004.pdf]
