# Supplementary material for: Genome-Wide Association Study to Identify the Genetic Determinants of Otitis Media Susceptibility in Childhood
Source: PLoS One. 2012 Oct 25;7(10):e48215. doi: 10.1371/journal.pone.0048215 (PMC3485007; doi:10.1371/journal.pone.0048215)
Supplement: Table S1 — Analysis in GenABEL showing the influence of adjustment for clinical and environmental covariates on associations with OM in 831 Raine Study participants for whom full covariate data were available. Data are shown for PCA adjustment only (Padj-PCA), and with additional adjustment for covariates including daycare attendance <3 yrs (Padj-daycare), allergy at < 3 yrs (Padj-allergy), non-exclusive breast feeding at <6 mths (Padj-supp), and all covariates (Padj-allCov). (PDF) [file pone.0048215.s005.pdf]

Table S1. Analysis in GenABEL showing the influence of adjustment for clinical and environmental covariates on associations with OM in 831 Raine Study participants for whom full covariate data were available. Data are shown for PCA adjustment only ($P_{adj\text{-}PCA}$), and with additional adjustment for covariates including daycare attendance <3 yrs ($P_{adj\text{-}daycare}$), allergy at < 3yrs ($P_{adj\text{-}allergy}$), non-exclusive breast feeding at <6 mths ($P_{adj\text{-}supp}$), and all covariates ($P_{adj\text{-}allCov}$). Key: Chr=chromosome, SNP=single nucleotide polymorphism, Position=bp position on Chr, A1/A2=alleles, N=number in analysis, effB= estimate of the β coefficient for each SNP, effBSE=standard error of effB, P1df=P-value for allele-wise test, OR=odds ratio, LL=lower 95% confidence interval, UL=upper 95% confidence interval. Estimates of effB (bold) for each SNP did not change appreciably across the analyses with adjustment for different covariates.

Children with covariate data, genotyped SNPs

Chr	SNP	Position	A1	A2	N	$P_{adj\text{-}PCA}$						$P_{adj\text{-}daycare}$						$P_{adj\text{-}allergy}$						$P_{adj\text{-}supp}$						$P_{adj\text{-}allcov}$						CONSEQUENCE	HGNC
						**effB**	effBSE	P1df	OR	LL	UL	**effB**	effBSE	P1df	OR	LL	UL	**effB**	effBSE	P1df	OR	LL	UL	**effB**	effBSE	P1df	OR	LL	UL	**effB**	effBSE	P1df	OR	LL	UL		
2	rs2098787	31150148	G	A	831	**0.46**	0.11	3.17E-05	1.58	1.28	1.97	**0.48**	0.11	1.63E-05	1.62	1.30	2.02	**0.47**	0.11	2.10E-05	1.61	1.29	2.00	**0.48**	0.11	1.80E-05	1.61	1.30	2.01	**0.51**	0.11	7.26E-06	1.66	1.33	2.08	INTRONIC	GALNT14
2	rs1862981	31151028	C	A	831	**0.47**	0.11	2.20E-05	1.60	1.29	1.99	**0.49**	0.11	1.10E-05	1.64	1.31	2.04	**0.48**	0.11	1.46E-05	1.62	1.30	2.02	**0.49**	0.11	1.29E-05	1.63	1.31	2.03	**0.52**	0.11	5.10E-06	1.68	1.34	2.10	INTRONIC	GALNT14
2	rs13408922	31298330	C	A	831	**0.63**	0.17	2.31E-04	1.87	1.34	2.62	**0.63**	0.17	2.14E-04	1.88	1.35	2.64	**0.59**	0.17	6.60E-04	1.80	1.28	2.52	**0.67**	0.17	1.01E-04	1.96	1.39	2.74	**0.64**	0.17	2.78E-04	1.88	1.34	2.65	NMD_TRANS	CAPN14
2	rs13386745	31299119	A	G	831	**0.62**	0.17	2.93E-04	1.85	1.33	2.58	**0.62**	0.17	2.60E-04	1.87	1.33	2.61	**0.57**	0.17	8.12E-04	1.78	1.27	2.49	**0.66**	0.17	1.24E-04	1.94	1.38	2.71	**0.63**	0.17	2.39E-04	1.90	1.35	2.67	NMD_TRANS	CAPN14
2	rs13386850	31299195	A	C	831	**0.63**	0.17	2.31E-04	1.87	1.34	2.62	**0.63**	0.17	2.14E-04	1.88	1.35	2.64	**0.59**	0.17	6.60E-04	1.80	1.28	2.52	**0.67**	0.17	1.01E-04	1.96	1.39	2.74	**0.64**	0.17	4.26E-03	2.19	1.28	3.74	NMD_TRANS	CAPN14
3	rs17624623	61595506	A	G	830	**0.47**	0.11	2.02E-05	1.60	1.29	1.98	**0.46**	0.11	3.26E-05	1.58	1.27	1.96	**0.46**	0.11	3.38E-05	1.58	1.27	1.96	**0.47**	0.11	2.47E-05	1.59	1.28	1.98	**0.45**	0.11	5.67E-05	1.57	1.26	1.95	INTRONIC	PTPRG
7	rs10242197	90097848	G	A	831	**-0.61**	0.15	4.84E-05	0.54	0.41	0.73	**-0.59**	0.15	9.79E-05	0.56	0.41	0.75	**-0.61**	0.15	5.01E-05	0.54	0.40	0.73	**-0.62**	0.15	4.23E-05	0.54	0.40	0.72	**-0.60**	0.15	7.81E-05	0.55	0.41	0.74	INTRONIC	CDK14
8	rs2882460	62688450	C	A	831	**0.51**	0.12	4.96E-05	1.66	1.30	2.12	**0.50**	0.13	7.57E-05	1.64	1.28	2.10	**0.49**	0.13	9.98E-05	1.63	1.27	2.08	**0.50**	0.13	5.55E-05	1.66	1.30	2.12	**0.48**	0.13	1.42E-04	1.62	1.26	2.07	INTRONIC	ASPH
8	rs6471969	62729386	C	A	831	**0.60**	0.13	3.85E-06	1.83	1.42	2.37	**0.59**	0.13	6.33E-06	1.81	1.40	2.34	**0.59**	0.13	6.86E-06	1.81	1.40	2.34	**0.61**	0.13	3.73E-06	1.84	1.42	2.38	**0.59**	0.13	9.38E-06	1.80	1.39	2.34	INTRONIC	ASPH
8	rs11990408	62747549	A	G	831	**0.57**	0.13	1.10E-05	1.76	1.37	2.27	**0.56**	0.13	1.85E-05	1.74	1.35	2.25	**0.56**	0.13	1.85E-05	1.74	1.35	2.25	**0.57**	0.13	1.21E-05	1.77	1.37	2.28	**0.55**	0.13	2.92E-05	1.73	1.34	2.24	INTRONIC	ASPH
8	rs11787089	62783393	G	A	831	**0.63**	0.14	6.83E-06	1.88	1.43	2.47	**0.62**	0.14	1.00E-05	1.86	1.41	2.45	**0.61**	0.14	1.67E-05	1.83	1.39	2.42	**0.65**	0.14	4.54E-06	1.91	1.45	2.52	**0.62**	0.14	1.48E-05	1.85	1.40	2.45	INTRONIC	ASPH
10	rs16919668	20319164	A	G	829	**-0.69**	0.17	3.28E-05	0.50	0.36	0.70	**-0.71**	0.17	2.05E-05	0.49	0.35	0.68	**-0.68**	0.17	4.56E-05	0.51	0.37	0.70	**-0.70**	0.17	2.92E-05	0.50	0.36	0.69	**-0.70**	0.17	2.64E-05	0.49	0.36	0.69	INTRONIC	PLXDC2
13	rs1336708	101763004	A	G	831	**-0.56**	0.14	5.17E-05	0.57	0.44	0.75	**-0.55**	0.14	6.67E-05	0.58	0.44	0.76	**-0.57**	0.14	3.31E-05	0.56	0.43	0.74	**-0.54**	0.14	8.73E-05	0.58	0.45	0.76	**-0.55**	0.14	7.20E-05	0.58	0.44	0.76	INTRONIC	FGF14
13	rs4512966	109880059	G	A	830	**0.44**	0.11	5.01E-05	1.55	1.25	1.91	**0.43**	0.11	7.18E-05	1.53	1.24	1.90	**0.43**	0.11	7.08E-05	1.53	1.24	1.90	**0.46**	0.11	2.52E-05	1.58	1.28	1.95	**0.44**	0.11	4.91E-05	1.56	1.26	1.93	INTRONIC	COL4A2
15	rs6493973	56079928	G	A	831	**1.23**	0.30	4.40E-05	3.43	1.90	6.20	**1.22**	0.30	5.81E-05	3.38	1.87	6.13	**1.23**	0.30	4.94E-05	3.43	1.89	6.22	**1.22**	0.30	5.55E-05	3.40	1.88	6.17	**1.21**	0.31	7.53E-05	3.36	1.84	6.13	INTRONIC	ALDH1A2
15	rs2218261	56081314	G	A	831	**1.23**	0.30	4.40E-05	3.43	1.90	6.20	**1.22**	0.30	5.81E-05	3.38	1.87	6.13	**1.23**	0.30	4.94E-05	3.43	1.89	6.22	**1.22**	0.30	5.55E-05	3.40	1.88	6.17	**1.21**	0.31	7.53E-05	3.36	1.84	6.13	INTRONIC	ALDH1A2
15	rs2704219	56115740	A	G	831	**1.22**	0.30	3.72E-05	3.39	1.90	6.05	**1.20**	0.30	5.33E-05	3.32	1.86	5.95	**1.22**	0.30	4.44E-05	3.37	1.88	6.04	**1.23**	0.30	3.95E-05	3.41	1.90	6.11	**1.20**	0.30	6.09E-05	3.33	1.85	6.01	INTRONIC	ALDH1A2
17	rs4627412	76138839	A	G	831	**0.46**	0.11	4.92E-05	1.58	1.27	1.96	**0.46**	0.11	4.63E-05	1.58	1.27	1.97	**0.45**	0.11	6.82E-05	1.57	1.26	1.95	**0.45**	0.11	5.64E-05	1.58	1.26	1.97	**0.45**	0.11	7.70E-05	1.57	1.25	1.96	INTRONIC	RPTOR
17	rs9911978	76139002	A	G	831	**0.46**	0.11	4.92E-05	1.58	1.27	1.96	**0.46**	0.11	4.63E-05	1.58	1.27	1.97	**0.45**	0.11	6.82E-05	1.57	1.26	1.95	**0.45**	0.11	5.64E-05	1.58	1.26	1.97	**0.45**	0.11	7.70E-05	1.57	1.25	1.96	INTRONIC	RPTOR
20	rs17396317	31254038	G	A	831	**0.59**	0.15	1.25E-04	1.81	1.34	2.45	**0.60**	0.16	1.21E-04	1.82	1.34	2.46	**0.60**	0.16	1.27E-04	1.81	1.34	2.46	**0.63**	0.16	5.87E-05	1.87	1.38	2.54	**0.63**	0.16	6.51E-05	1.88	1.38	2.56	WITHIN_pseudogene	BPIFA4P
20	rs17305657	31270249	A	G	831	**0.68**	0.17	8.64E-05	1.98	1.41	2.78	**0.69**	0.17	8.70E-05	1.99	1.41	2.80	**0.70**	0.18	6.95E-05	2.01	1.42	2.83	**0.73**	0.18	3.56E-05	2.07	1.47	2.92	**0.74**	0.18	3.28E-05	2.10	1.48	2.97	INTRONIC	BPIFA3
20	rs3818222	31276584	A	G	831	**-0.38**	0.11	5.47E-04	0.68	0.55	0.85	**-0.40**	0.11	4.12E-04	0.67	0.54	0.84	**-0.38**	0.11	7.64E-04	0.69	0.55	0.85	**-0.38**	0.11	5.86E-04	0.68	0.55	0.85	**-0.39**	0.11	3.28E-05	0.68	0.54	0.85	NON_SYN_CODING	BPIFA3
20	rs6059169	31281265	A	C	831	**0.42**	0.12	3.88E-04	1.53	1.21	1.93	**0.43**	0.12	3.60E-04	1.53	1.21	1.94	**0.41**	0.12	5.31E-04	1.51	1.20	1.91	**0.44**	0.12	2.33E-04	1.56	1.23	1.97	**0.44**	0.12	6.04E-04	1.55	1.22	1.97	DOWNSTREAM	BPIFA1
20	rs6059183	31289688	A	G	831	**0.41**	0.12	5.32E-04	1.51	1.20	1.91	**0.42**	0.12	5.08E-04	1.52	1.20	1.92	**0.41**	0.12	7.03E-04	1.50	1.19	1.90	**0.43**	0.12	3.18E-04	1.54	1.22	1.95	**0.43**	0.12	3.01E-04	1.54	1.21	1.95	SPLICE_SITE,INTRONIC	BPIFA1
21	rs2839520	42742732	A	G	831	**-0.47**	0.11	1.63E-05	0.63	0.51	0.77	**-0.45**	0.11	3.60E-05	0.64	0.52	0.79	**-0.46**	0.11	2.01E-05	0.63	0.51	0.78	**-0.46**	0.11	3.00E-05	0.63	0.51	0.79	**-0.44**	0.11	6.75E-05	0.65	0.52	0.80	DOWNSTREAM	UBASH3A
