# Supplementary material for: Genome-Wide Association Study to Identify the Genetic Determinants of Otitis Media Susceptibility in Childhood
Source: PLoS One. 2012 Oct 25;7(10):e48215. doi: 10.1371/journal.pone.0048215 (PMC3485007; doi:10.1371/journal.pone.0048215)
Supplement: Table S2 — Directed interrogation of the Raine Study Discovery GWAS results across genes/regions previously implicated in childhood OM susceptibility in the literature: (A) candidate genes previously reported to be associated with OM susceptibility; (B) the human homologues of genes identified from murine models of OM; and (C) regions of the human genome previously implicated in genome-wide linkage studies. For all genes/regions and SNPs the physical location is based on annotation from Ensembl release 54 with gene boundaries defined as +/- 20 kb from the 5′ and 3′ UTR. Results are presented from the discovery GWAS adjusted for PCs alone. This analysis was undertaken to find any evidence of association at these genes, not as a direct replication of specific SNPs reported in other studies. Nominal P<0.05 are highlighted in bold. No associations were robust to correction for the number of genes interrogated under each hypothesis (see main text). (PDF) [file pone.0048215.s006.pdf]

Table S2. Directed interrogation of the Raine Study Discovery GWAS results across genes/regions previously implicated in childhood OM susceptibility in the literature: (A) candidate genes previously reported to be associated with OM susceptibility; (B) the human homologues of genes identified from murine models of OM; and (C) regions of the human genome previously implicated in genome-wide linkage studies. For all genes/regions and SNPs the physical location is based on annotation from Ensembl release 54 with gene boundaries defined as +/- 20kb from the 5` and 3` UTR. Results are presented from the discovery GWAS adjusted for PCs alone. This analysis was undertaken to find any evidence of association at these genes, not as a direct replication of specific SNPs reported in other studies. Nominal P<0.05 are highlighted in bold. No associations were robust to correction for the number of genes interrogated under each hypothesis (see main text).

(A)

Gene	Chr (Start - Finish)	Top GWAS P-value	Top GWAS SNP	SNP Physical Location	SNP Position	Strand
**Raine Study discovery GWAS results - PCA adjusted**						
IL10	1 (205137412 - 205144107)	**0.0098**	**rs3021094**	205011575	Intronic	Reverse
FBXO11	2 (47887563 - 47986318)	**4.64×10^{-5}**	**rs330787**	47894881	Intronic	Reverse
IL1A	2 (113247963 - 113259442)	0.2458	rs6746923	113269897	Intergenic	Reverse
IL1B	2 (113303808 - 113310827)	0.2143	rs3917368	113299253	Downstream	Reverse
CD14	5 (139991501 - 139993439)	0.5380	rs778591	140005668	Intronic	Reverse
HLA-A	6 (30018305 - 30021633)	0.3350	rs16896742	30030719	Intergenic	Forward
TNF	6 (31651329 - 31654091)	**0.0354**	**rs2229092**	31648736	Non Synonymous (H-P)	Forward
IL6	7 (22733323 - 22738145)	0.1393	rs10499563	22727013	Downstream	Forward
SERPINE1	7 (100557099 - 100569262)	0.4880	rs2227631	100556258	Upstream	Forward
TLR4	9 (119506431 - 119519587)	0.1949	rs2737191	119502536	Upstream	Forward
MBL2	10 (54195146 - 54201466)	0.1458	rs1800450	54201241	Non Synonymous (G-D)	Reverse
SFTPA1/SFTPA2	10 (81040722 - 81045208)	0.6163	rs1650169	810584202	Intergenic	Forward
SFTPD	10 (81687476 - 81698841)	**0.0331**	**rs1923539**	81684930	Intronic	Reverse
MUC2	11 (1064875 - 1094417)	**0.0033**	**rs7934606**	1083945	Intronic	Forward
MUC5AC/MUC5B	11 (1132474 - 1245302)	0.1252	rs6421966	1116979	Intergenic	Forward
IFNG	12 (66834817 - 66839788)	0.5699	rs12369470	66831163	Downstream	Reverse
SMAD2	18 (43613464 - 43711510)	0.2409	rs4940086	43700305	Intronic	Reverse
SMAD4	18 (46810581 - 46865409)	0.2249	rs12958604	46814223	Intronic	Forward
SCN1B	19 (40213374 - 40223193)	0.1103	rs2278996	40222621	3' untranslated region	Forward
TGFB1	19 (46528491 - 46551656)	0.3621	rs10417924	46525007	Downstream	Reverse

For details of original publications see Rye *et al.* [11]

(B)

Mouse Gene	Human Gene	Chr (Start - Finish)	Top GWAS P-value	Top GWAS SNP	SNP Physical Location	SNP Position	Strand
**Raine Study discovery GWAS results - PCA adjusted**							
P73	TP73	1 (3558989 - 3640327)	**0.0112**	**rs3765766**	3624520	Intronic	Forward
Fbxo11	FBXO11	2 (47887563 - 47986318)	**$4.64x10^{-5}$**	**rs330787**	47894881	Intronic	Reverse
Evi1	EVI1	3 (170283981 - 170348216)	0.1147	rs9809990	170354088	Intergenic	Reverse
MyD88	MYD88	3 (38155009 - 38159516)	0.1796	rs7744	38159025	3' untranslated	Forward
Idua	IDUA	4 (970785 - 988317)	0.2556	rs3822030	977343	Intronic	Forward
Tlr2	TLR2	4 (154824891 - 154846692)	**0.0442**	**rs7656411**	154847105	Downstream	Forward
Dnahc5	DNAH5	5 (13743437 - 13997589)	**0.0151**	**rs17265607**	13867353	Intronic	Reverse
Isl1	ISL1	5 (50714715 - 50726320)	**0.0464**	**rs6449600**	50711054	Upstream	Forward
Sh3pxd2b	SH3PXD2B	5 (171693108 - 171814132)	**0.0130**	**rs2731693**	171808689	Intronic	Reverse
Eya4	EYA4	6 (133604188 - 133894951)	0.1204	rs12110683	133896363	Downstream	Forward
Plg	PLG	6 (161043264 - 161094337)	0.1039	rs813641	161073980	Intronic	Forward
Dnahc11	DNAH11	7 (21549358 - 21907982)	**0.0162**	**rs933353**	21745629	Intronic	Forward
Gus	GUSB	7 (65063108 - 65084681)	0.4429	rs4718296	65073571	Intronic	Reverse
Fgfr1	FGFR1	8 (38387813 - 38445509)	0.0302	rs13317	38388671	3' untranslated	Reverse
Tlr4	TLR4	9 (119506431 - 119519587)	0.1949	rs2737191	119502536	Upstream	Forward
Fas	FAS	10 (90740268 - 90765522)	**0.0205**	**rs12765241**	90730481	Intergenic	Forward
IκBαδN	NFKBIA	14 (34940467 - 34943711)	0.1520	rs3138045	34947472	Upstream	Reverse
E2f4	E2F4	16 (65783569 - 65790322)	0.1203	rs868213	65777958	Intergenic	Forward
Naglu	NAGLU	17 (37941477 - 37949992)	0.6003	rs647397	37932924	Downstream	Forward
Sall4	SALL4	20 (49833990 - 49852455)	0.2496	rs6096597	49870980	Intergenic	Reverse
Cby1	CBY1	22 (37382604 - 37399801)	0.7905	rs5757213	37383834	Intronic	Forward
Nf2	NF2	22 (28329545 - 28424589)	0.0538	rs9614025	28383939	Intronic	Forward
Tbx1	TBX1	22 (18124226 - 18134855)	0.1226	rs9618678	18104613	Intergenic	Reverse
Ids	IDS	X (148368203 - 148394769)	0.0943	rs584058	148412774	Intergenic	Reverse
Phex	PHEX	X (21960842 - 22176399)	0.0526	rs178172	21963292	Intronic	Forward

For details of original publications see Rye *et al.* [32]

(C)

Region	Chr (Start - Finish)	Top GWAS P-value	Top GWAS Gene	Top GWAS SNP	SNP Physical Location	SNP Position	Strand
**Raine Study discovery GWAS results - PCA adjusted**							
3p25.3	3 (8700001 - 11500000)	**0.0028**	SRGAP3	rs383563	Intronic	9027494	Reverse
		**0.0011**	ATP2B2	rs741498	Intronic	10500607	Reverse
10q22.3	10 (77400001 - 82000000)	**0.0014**	Intergenic	rs1665568	Intergenic	80084189	N/A
10q26.3	10 (130500001 - 135374737)	**0.0022**	PPP2R2D	rs7072006	Upstream	133595618	Forward
17q12	17 (28800001 - 35400000)	**$3.76x10^{-4}$**	SLFN5	rs1564580	Intergenic	30587907	Forward
19q13.4	19 (59100001 - 63811651)	**0.0017**	LILRA2	rs11672845	Upstream	59773745	Forward

For details of original publications see Daly *et al.* [8] and Casselbrant *et al.* [9]; N/A - no strand information available for intergenic SNPs.
