# Supplementary material for: Genome-Wide Association Study to Identify the Genetic Determinants of Otitis Media Susceptibility in Childhood
Source: PLoS One. 2012 Oct 25;7(10):e48215. doi: 10.1371/journal.pone.0048215 (PMC3485007; doi:10.1371/journal.pone.0048215)
Supplement: Table S3 — SNPs/Genes selected for replication analysis in the WAFSOM cohort. (PDF) [file pone.0048215.s007.pdf]

Table S3. SNPs/Genes selected for replication analysis in the WAFSOM cohort.

SNP	Gene	Chr	Position	Major/ Minor	Ancestral Allele
rs1862981	GALNT14	2	31151028	C/A	C
rs2113490	GALNT14	2	31157651	A/G	A
rs12621279	GALNT14	2	31195804	C/T	C
rs17010928	CAPN14	2	31268558	G/A	G
rs6728152	CAPN14	2	31285155	T/C	T
rs13408922	CAPN14	2	31298330	C/A	A
rs11684139	GALNT13	2	154725951	T/G	T
rs707069	GALNT13	2	155005699	T/C	T
rs799822	GALNT13	2	155007126	G/A	A
rs16887121	BMP5	6	55746041	A/G	G
rs10456718	BMP5	6	55768837	T/C	T
rs1470527	BMP5	6	55819170	C/T	T
rs11025841	NELL1	11	20948783	A/C	A
rs1914243	NELL1	11	20971341	A/C	A
rs1945331	NELL1	11	21220418	C/T	C
rs3917192	TGFB3	14	75501427	G/A	G
rs2284792	TGFB3	14	75513332	A/G	G
rs2268626	TGFB3	14	75514520	T/C	T
rs6059183	BPIFA1	20	31289688	T/C	T
rs6059187	BPIFA1	20	31291926	G/A	G
