# Supplementary material for: Genome-Wide Association Study to Identify the Genetic Determinants of Otitis Media Susceptibility in Childhood
Source: PLoS One. 2012 Oct 25;7(10):e48215. doi: 10.1371/journal.pone.0048215 (PMC3485007; doi:10.1371/journal.pone.0048215)
Supplement: Table S4 — Replication of selected genes in the WAFSOM cohort using case/pseudo-control conditional logistic regression under an additive model. Where possible, tag-SNPs were selected to match SNPs genotyped in the Raine cohort. For some genes we selected additional tag-SNPs to cover areas of the gene not well covered on the SNP-chip used to analyse Raine. Padj-PCA values from the Raine cohort are provided for ease of comparison. (PDF) [file pone.0048215.s008.pdf]

Table S4. Replication of selected genes in the WAFSOM cohort using case/pseudo-control conditional logistic regression under an additive model. Where possible, tag-SNPs were selected to match SNPs genotyped in the Raine cohort. For some genes we selected additional tag-SNPs to cover areas of the gene not well covered on the SNP-chip used to analyse Raine. $P_{adj\text{-}PCA}$ values from the Raine cohort are provided for ease of comparison.

SNP	Chr	Major/ Minor	MAF	Risk Allele	Robust P-Value	Odds Ratio[a] (95% CI)	Raine P_{adjPCA}
GALNT14_rs1862981	2	C/A	0.45	C	0.412	1.06 (0.92 - 1.23)	0.0036
GALNT14_rs2113490	2	A/G	0.46	G	0.653	1.03 (0.89 - 1.19)	0.0018
GALNT14_rs12621279	2	C/T	0.36	C	0.502	1.06 (0.90 - 1.25)	0.0055
CAPN14_rs17010928	2	G/A	0.08	G	0.522	1.10 (0.82 - 1.49)	9.85×10^{-5}
CAPN14_rs6728152	2	T/C	0.04	T	0.058	1.43 (0.99 - 2.07)	1.75×10^{-4}
CAPN14_rs13408922	2	C/A	0.10	C	0.092	1.23 (0.97 - 1.57)	1.32×10^{-6}
GALNT13_rs11684139	2	T/G	0.25	T	0.759	1.03 (0.86 - 1.24)	0.0023
GALNT13_rs707069	2	T/C	0.12	T	0.223	1.16 (0.91 - 1.47)	0.0069
GALNT13_rs799822	2	G/A	0.08	G	0.132	1.24 (0.94 - 1.65)	-
BMP5_rs16887121	6	A/G	0.07	A	0.811	1.04 (0.77 - 1.39)	0.007
BMP5_rs10456718	6	T/C	0.23	C	0.755	1.03 (0.87 - 1.22)	-
BMP5_rs1470527	6	C/T	0.40	T	0.378	1.07 (0.92 - 1.24)	0.0031
NELL1_rs11025841	11	A/C	0.09	A	0.540	1.08 (0.84 - 1.39)	-
NELL1_rs1914243	11	A/C	0.18	N/A	1.000	1.00 (0.82 - 1.22)	-
NELL1_rs1945331	11	C/T	0.17	T	0.222	1.13 (0.93 - 1.38)	0.0027
TGFB3_rs3917192	14	G/A	0.16	A	0.278	1.12 (0.91 - 1.37)	0.0022
TGFB3_rs2284792	14	A/G	0.26	N/A	1.000	1.00 (0.85 - 1.17)	-
TGFB3_rs2268626	14	T/C	0.20	C	0.330	1.09 (0.91 - 1.31)	-
BPIFA1_rs6059183	20	T/C	0.26	N/A	1.000	1.00 (0.84 - 1.19)	6.72×10^{-4}
BPIFA1_rs6059187	20	G/A	0.49	A	0.511	1.05 (0.90 - 1.23)	-

Where MAF = minor allele frequency in WAFSOM, CI = 95% confidence intervals.

[a] **-** Odds ratios are shown for the risk allele.
